# Publisher Correction: npj Systems Biology and Applications, volume 5

**DOI:** 10.1038/s41540-019-0102-7

**Published:** 2019-08-13

**Authors:** npj Systems Biology and Applications

**Affiliations:** London, United Kingdom

An error occurred during the publication of a number of articles in *npj Systems Biology and Applications*. Several articles were published in volume 5 with a duplicate citation number.

In this correction article the old and new citation metadata are published in Table 1.

**Table 1:** Overview of incorrect and correct citation metadata

DOI	Published online date	Incorrect citation number	Correct citation number
10.1038/s41540-019-0097-0	14 June 2019	1	21

The original articles have been updated. The publisher apologises for the inconvenience caused to our authors and readers.

