# Supplementary material for: Combinatorial perturbation analysis reveals divergent regulations of mesenchymal genes during epithelial-to-mesenchymal transition
Source: NPJ Syst Biol Appl. 2019 Jun 14;5:21. doi: 10.1038/s41540-019-0097-0 (PMC6570767; doi:10.1038/s41540-019-0097-0)
Supplement: Supplementary file 1 — Supplementary Information [file 41540_2019_97_MOESM1_ESM.pdf]

SUPPLEMENTARY INFORMATION

SUPPLEMENTARY METHODS…………………………………………………………………………………………….4

Supplementary Method 1. Regulation of M-gene clusters by other EMT regulators……4

Supplementary Method 2. Gene set enrichment analysis of M-gene clusters………………7

Supplementary Method 3. Expression of M-gene clusters in mesenchymal-like cancer cell lines…….9

SUPPLEMENTARY FIGURES…………………………………………………………………………………………11

Supplementary Figure 1. Establishment of ZEB1-deficient MCF10A cells……………………11

Supplementary Figure 2. Role of ZEB1 in reciprocity of E and M phenotypes in EMT…..12

Supplementary Figure 3. Expression of EMT genes in the 8 combinations of TGF-β and ZEB1 treatments…………………………………………………………………………………………………..14

Supplementary Figure 4. Quantification of EMT gene expression in response to TGF-β and ZEB1 under different definitions of differential expression…………………………………………..15

Supplementary Figure 5. Clustering E- and M-genes according to log fold-change of expression……………………………………………………………………………………………………….17

Supplementary Figure 6. Elbow plot showing the changes of sum of squared errors as the number of clusters increases……………………………………………………………………………….18

Supplementary Figure 7. Principal Component Analysis of CAGE expression data for M-genes……19

Supplementary Figure 8. Comparison of expression patterns representative genes in four clusters using CAGE and RT-PCR……………………………………………………………………………20

Supplementary Figure 9. Expression of E-gene cluster under TGF-β and ZEB1 regulation………21

Supplementary Figure 10. Significance and confidence intervals for difference in odds of enrichment between M-gene groups across different regulatory data sets…………………22

Supplementary Figure 11. Log fold-change of expression of each M-gene cluster in response to GSC overexpression………………………………………………………………………………23

Supplementary Figure 12. Expression of three cluster of M-genes in cancer cell lines…24

Supplementary Figure 13. Positive correlations among M3 genes………………………………25

Supplementary Figure 14. Correlation between changes of mean gene expression and velocity of cells under 8 contrast conditions……………………………………………………………26

Supplementary Figure 15. Metaphoric illustration of relationship between reciprocity of E- and M-genes and irreversibility of EMT transition……………………………………………………27

SUPPLEMENTARY TABLES…………………………………………………………………………………………28

Supplementary Table 1. Overlapping EMT gene expression before and after annotating dbEMT genes………………………………………………………………………………………………………..28

Supplementary Table 2. List of EMT genes analyzed in this study and their clustering information………29

Supplementary Table 3. Enrichment of EMT genes among gene upregulated by EMT regulators in Tauble et al…………………………………………………………………………………………44

Supplementary Table 4. List of GO Terms enriched in all epithelial genes……………………45

Supplementary Table 5. List of GO Terms enriched in epithelial genes which are differentially expressed in response to both TGF-β and ZEB1……………………………………50

Supplementary Table 6. Gene sets enriched in M-gene clusters…………………………………51

Supplementary Table 7. Genes from M-gene clusters overlapping with enriched cancer gene sets ZEB1…………………………………………………………………………………………………..55

Supplementary Table 8. List of primer sequences………………………………………………….58

Supplementary Table 9. Parameter values for mathematical models…………….…………59

SUPPLEMENTARY REFERENCES…………………………………………………………………………………60

SUPPLEMENTARY METHODS

Supplementary Method 1 - Regulation of M-gene clusters by other EMT regulators

To further investigate the regulation of M-gene clusters, we employed microarray expression data generated by Taube et al. [1] for four transcription factors identified as being upstream EMT inducers (TGF-β, Snail, Twist, and Gsc) as well as knockdown of E-cadherin (sh-Ecad). Taube et al. reported as list of 159 genes down-regulated under all conditions and 87 up-regulated under all conditions. We found that E-genes are significantly enriched among down-regulated genes (55 downregulated; Fisher's exact test, p = 6.6e-52) and M-genes are significantly enriched among up-regulated genes (37 upregulated, Fisher's exact test, p = 1.1e-36). However, because of the strict selection criteria, these genes are highly expressed across all condition and there are relatively few of them in each cluster (18, 15 and 4 in M1, M2, and M3 respectively). As such, we were unable to use these upregulated genes alone to distinguish between our M-gene clusters.

Therefore, we reanalyzed the microarray data produced by Taube et al. [1] Data relating to the Taube et al. study was retrieved from the Gene Expression Omnibus, accession numbers GSE9691 and GSE24202. These data were analyzed using the 'limma' and 'affy' packages available through Bioconductor [2]. Normalization was done using RMA and the significance of each differential expression model (treatment – control) was determined using empirical Bayes. We applied the same criteria used by Taube et al. [1] (magnitude of fold-change > 2) to determine differential expression in response to each treatment. Enrichment of E, M, M1, M2, and M3-genes were evaluated using Fisher's exact test. Significance of enrichment differences between groups was determined using the log of the odds of ratio of enrichment, which is approximately normally distributed with a standard error of:

$$SE = \sqrt{\frac{1}{n_1} + \frac{1}{n_2} + \frac{1}{n_3} + \frac{1}{n_4}}$$

Where the n values are the counts of the contingency table used to determine enrichment.

Using this approach, we found that only E-genes are enriched among down-regulated genes for any factor, while both M-genes as a whole and each cluster of M-genes are enriched among in every set of up-regulated genes (**Supplementary Table 3**). To compare responsiveness across M-gene clusters, we looked at the difference in the log-odds of enrichment **(Supplementary Figure 10)** and found no evidence for differential enrichment for Snail, Twist, or E-cadherin knockdown. For TGF-β, we observe a non-significant reduction in the log-odds of enrichment for M3-genes, which we found to be significantly less responsive to TGF-β in our CAGE data set. However, we do not consider this a contradiction, given both differences methodology and the fact that, while not significant, the confidence interval of the difference of log-odds is biased in favor of reduced response to TGF-β. Conversely, there is evidence of differential response to Gsc between our M-genes clusters. There is a significant difference in the enrichment of Gsc up-regulated genes between M1 and M2 and a large but non-significant difference between M1 and M3, both of which indicate reduced enrichment of M1-genes among Gsc up-regulated genes. As such, it is not surprising that, when we considered the expression of all M-genes, we see a significant difference in the log fold-change of expression in response to Gsc between M1 and M2 genes (Mann-Whitney U-test, $p = 0.004$) and between M1 and M3 genes (Mann-Whitney U-test, $p = 0.019$), but not between M2 and M3 genes (Mann-Whitney U-test, $p = 0.5237$) (**Supplementary Figure 11**). Gsc is notable in that Taube et al. found it was not up-regulated by any of the other EMT inducers used in their study, suggesting that Gsc regulates EMT in an independent fashion. As

such, while these results indicate that M2 and M3 genes may be regulated by additional EMT factors, it is in a way that is independent of the TGF-β/ZEB1 pathway explored in our study. Together with our own loss-of-function and gain-of-function experiments, these analyses suggest that perturbations of single EMT factors may have limited and differential roles in EMT, which involves changes in high-dimensional gene expression space in the nonlinear fashion [3].

Supplementary Method 2 – Gene set enrichment analysis of M-gene clusters

Genes in each M-gene clusters were compared against gene set derived from gene set enrichment analysis in the Molecular Signature Database maintained by the Broad Institute [4]. Specifically, we compare M-gene clusters to selected cancer related expression sets from the 'CGP: chemical and genetic perturbations' database. We also considered the 'CGN: cancer gene neighborhoods', 'CM: cancer modules' and 'C6: oncogenic signatures' sets, but chose not to use them for the following reasons: the CGN and CM database contained mostly computationally clustered gene sets without further annotation while C6 focused response to one specific gene or factor in a cancer context rather differential expression across broad cancer phenotypes. Using the described criteria, we found 18, 16, and 22 sets enriched in M1, M2, and M3-gene clusters respectively (**Supplementary Table 6**) for a total 31 unique enriched sets, the majority (16) of which are enriched in more than one cluster. Of the remaining 15 sets, 10 are unique to M3 genes. The gene sets enriched in M-gene clusters can be used to distinguish between specific subtypes of cancer: for example, M3 genes are enriched amongst those up-regulated in luminal A breast cancer, but down-regulated in luminal B. M3 genes are also enriched in those up-regulated in papillary thyroid carcinoma but down regulated in follicular carcinoma. The specific genes involved in these cancers are listed in **Supplementary Table 7**. Nevertheless, we should be careful not to extrapolate the annotation to the whole of any M-gene cluster as the size of the enriched groups vary widely (between 3 and 41 genes, or 4% and 28% of a cluster). Furthermore, some annotations are contradictory, such as both M1 and M3 being enriched for genes that are up-regulated and down-regulated in basal breast cancer. Instead, these results suggest that M-

gene clusters contain sub-clusters of genes which are associated with a diversity cancer types, with M3 genes standing out as having both the greatest overall and most unique associations.

Supplementary Method 3. Expression of M-gene clusters in mesenchymal-like cancer cell lines

To examine the expression of M-gene clusters in cancer cells, we obtained RNA-sequencing data from the Cancer Cell Line Encyclopedia, which contains transcriptomic information of 1019 cancer cell lines [5]. We selected the cell lines with mesenchymal-like expression profile by calculating the mean M-genes and mean E-genes (the classification of E and M genes is based on our SOM followed by supervised learning. See **Methods**) expressions in all cell lines. Visual inspection of the expression profiles of the cells suggests that there is a large group of cancer cell lines that highly express M-genes but not E-genes (**Supplementary Figure 12A**). We used a simple threshold to select the mesenchymal-like cancer cell lines (mean M-gene log_2(RPKM) > -1, mean E-gene log_2(RPKM) < 1), and we obtained 416 such cell lines. We found that the mean expressions of the three M clusters are significantly different in these cell lines (**Supplementary Figure 12B**, p-values < 10^{-5}). The mean expression of M2 gene cluster is higher than that of the other two clusters by more than 2-fold, and the mean expression of M3 cluster is higher than that M1 cluster by 30% (**Supplementary Figure 12B**).

Because we showed that the high expression of M3 genes are significantly associated with good prognosis, we asked if these genes are correlated in cancer settings. We calculated the Pearson correlation coefficients of all pairs of M3 genes (within-cluster correlations) in terms of their expression in the 416 cell lines (**Supplementary Figure 12C**, red), and we compared these correlations with those of M3 genes with M1 and M2 genes (between-cluster correlations) (**Supplementary Figure 12C**, gray). We found that the within-cluster correlations are significantly more positive than the between-cluster correlations (0.020 vs. -0.008, $p < 10^{-5}$). However, the within-cluster correlations exhibit a large degree of heterogeneity (**Supplementary Figure 12C**),

reflecting the complexity of the regulations on these genes in cancer cells. Nonetheless, when we used a threshold (r > 0.25, above the maximal correlation in between-cluster gene pairs) to select genes that are involved in positive correlations. 73 out of 77 M3 genes were involved in the single cluster connected by edges based on the correlations (**Supplementary Figure 13**). Collectively, these results suggest that even though the regulatory circuits of M-gene clusters obtained in our study cannot be extrapolated to gene expressions in cancer cells directly, their differential patterns and within-cluster correlations are observed in cancer cells. Therefore, our study provides useful information in understanding M-genes in cancer cells.

SUPPLEMENTARY FIGURES

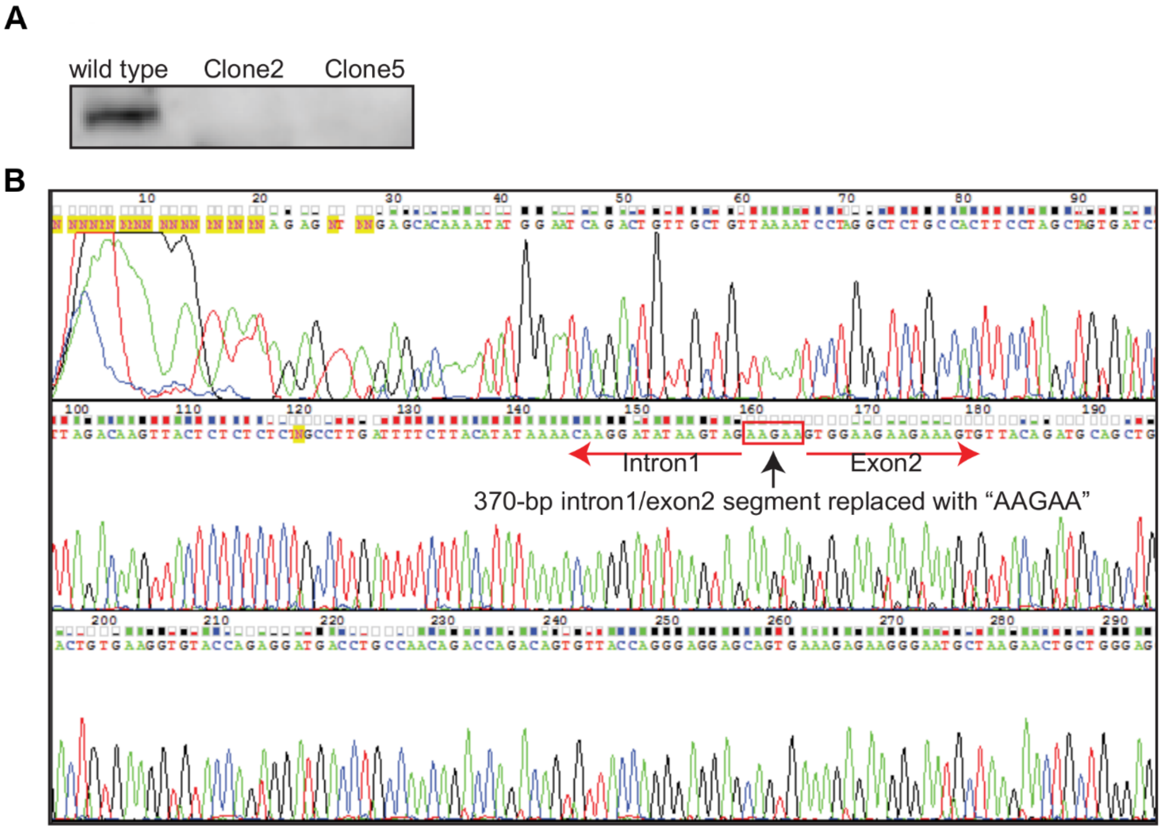

Supplementary Figure 1. Establishment of ZEB1-deficient MCF10A cells. (A) Western blotting for ZEB1 protein. Control MCF10A and ZEB1 KO clones were treated with TGF-β to induce endogenous ZEB1. **(B)** Sanger sequencing result at the Intron1-Exon2 boundary of ZEB1 gene in clone5.

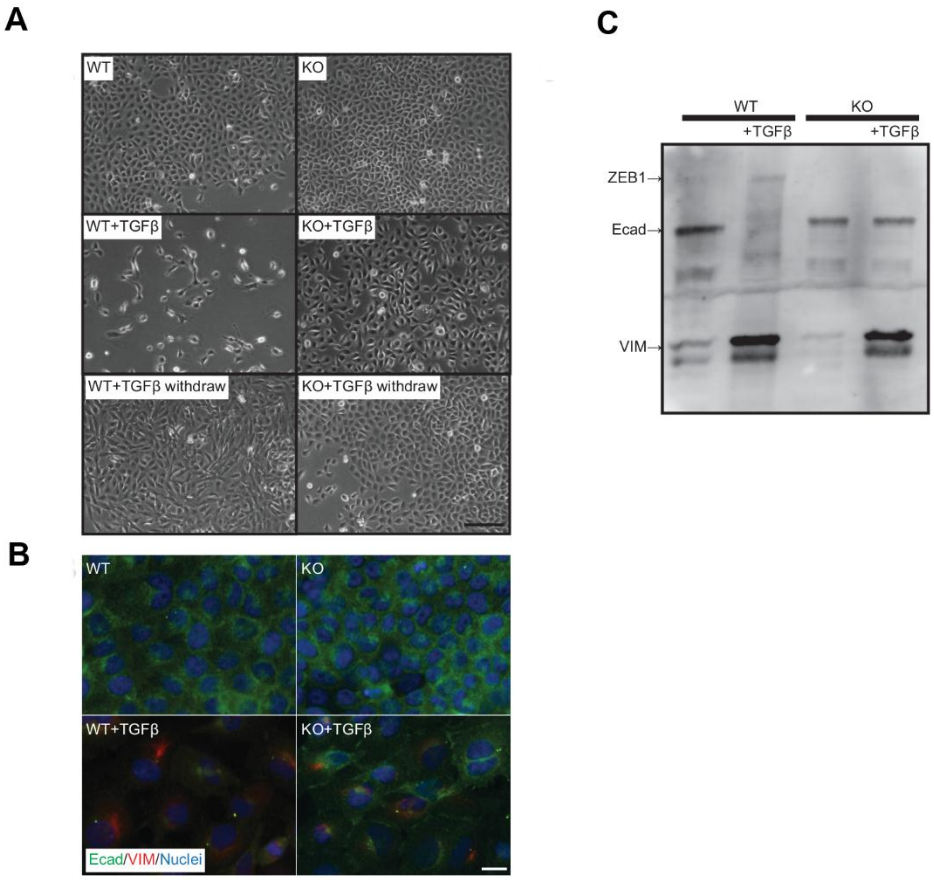

Supplementary Figure 2. Role of ZEB1 in reciprocity of E and M phenotypes in EMT. (A) Morphological changes induced by TGFβ treatment in wild-type (WT) and ZEB1-deficient (KO) cells. Cells were treated with 200 pM TGF-β recombinant protein for 2 weeks (+TGF-β) and then cultured without TGF-β for another 2 weeks (+TGF-β withdraw). Scale bar = 100 µm. **(B)** Immunofluorescent analysis of E-cadherin (Ecad, green) and vimentin (VIM, red). Cells were treated with 200 pM TGF-β recombinant protein for 2 weeks (+TGF-β). Nuclei were counterstained with DAPI. Scale bar = 20 µm. **(C)** Western blot analysis for epithelial (E) and mesenchymal (M) marker proteins. Protein samples were collected from cells after TGFβ treatment as described above. The blot was sequentially incubated with antibodies against the

indicated proteins (ZEB1, Ecad, and VIM, respectively) and the signals were detected simultaneously.

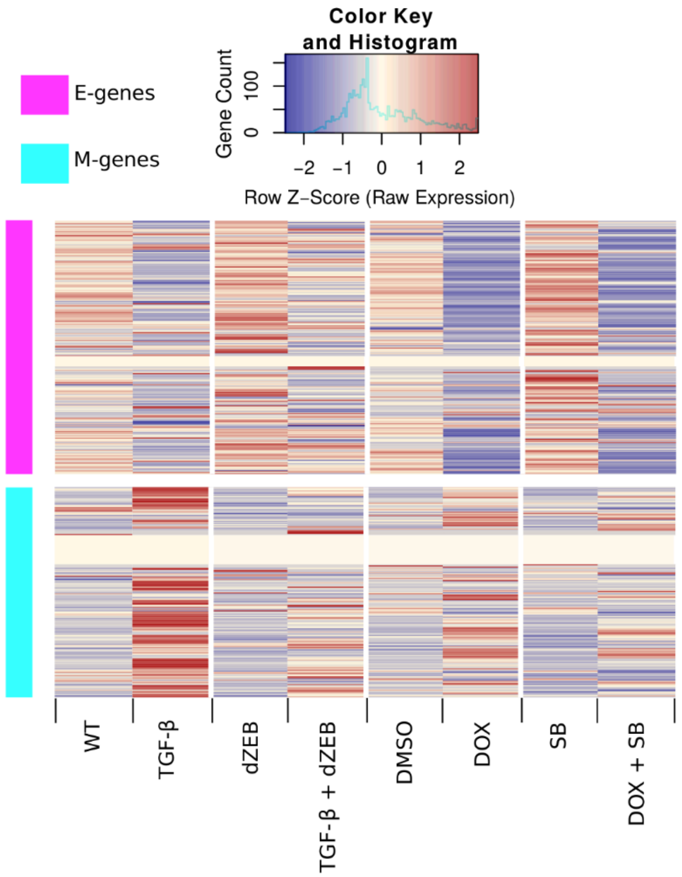

Supplementary Figure 3. Expression of EMT genes in the 8 combinations of TGF-β and ZEB1 treatments (see **Table 1**). Expression values were scaled across genes (rows) to adjust for different average expression between genes. Cells in the heatmap are colored based on the z-score of each expression value in its respective row, indicating whether the gene is expressed relatively higher (red) or lower (blue) in that condition than on average. E-genes (pink) and M-genes (light blue) were manually separated, then ordered using hierarchical clustering.

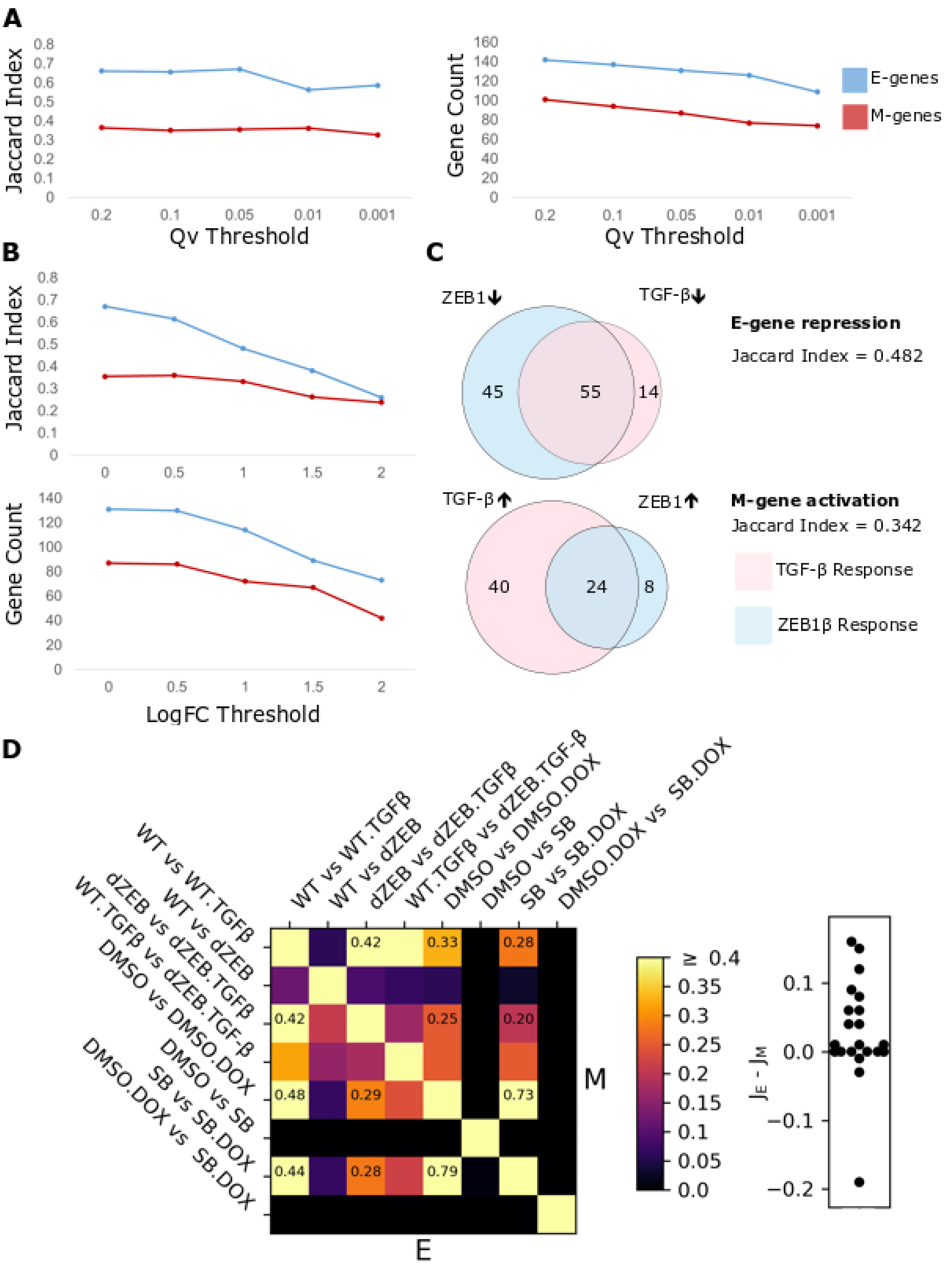

A
Jaccard Index
Qv Threshold
Gene Count
Qv Threshold
E-genes
M-genes
B
Jaccard Index
Gene Count
LogFC Threshold
C
ZEB1
TGF-β
45
55
14
E-gene repression
Jaccard Index = 0.482
TGF-β
ZEB1
40
24
8
M-gene activation
Jaccard Index = 0.342
TGF-β Response
ZEB1β Response
D
WT vs WT.TGFβ
WT vs dZEB
dZEB vs dZEB.TGFβ
WT.TGFβ vs dZEB.TGF-β
DMSO vs DMSO.DOX
DMSO vs SB
SB vs SB.DOX
DMSO.DOX vs SB.DOX
M
E
≥ 0.4
$J_E - J_M$

Supplementary Figure 4. Quantification of EMT gene expression in response to TGF-β and ZEB1 under different definitions of differential expression. (A) (Left) Jaccard index for response to TGF-β and ZEB1 using different q-value thresholds and (Right) the total number of genes passing the threshold for E-(blue) and M-(red) genes. **(B)** (Top) Jaccard index for response to TGF-β and ZEB1 using different log fold-change thresholds with q-value cutoff of 0.05 and (Bottom) the total number of genes passing both thresholds for E-(blue) and M-(red) genes. **(C)** Venn diagrams showing the overlap in E- (top) and M-(bottom) genes which show significant expression differences in response to TGF-β or ZEB1 treatment relative to their respective control conditions and have an absolute log fold-change >1. TGF-β response is in pink while ZEB1 response is in light blue. The number E- and M-genes responding to TGF-β or ZEB1 uniquely as well as those responding to both are listed in the respective part of the Venn diagram. The type of response, activation or repression, is indicated by directional arrows (up-arrow: activation, down-arrow: repression). The overlap was quantified using the Jaccard index, which is the number of genes differentially expressed genes by both TGF-β and ZEB1 divided by the total number of differentially expressed gene. **(D)** Jaccard indices of E- and M-genes for each pair of conditions where only genes with an absolute log fold-change >1 are considered. Heatmap shows all the Jaccard indices. Lower triangular entries: E-genes. Upper triangular entries: M genes. The Jaccard index those cells labeled in Figure 1d are also labeled here for comparison. Swarm plot shows the differences between the Jaccard indices of E-genes and those of the M-genes for each of the 28 pairs of conditions ($p < 0.001$ for single value t-test with a null distribution centered at 0).

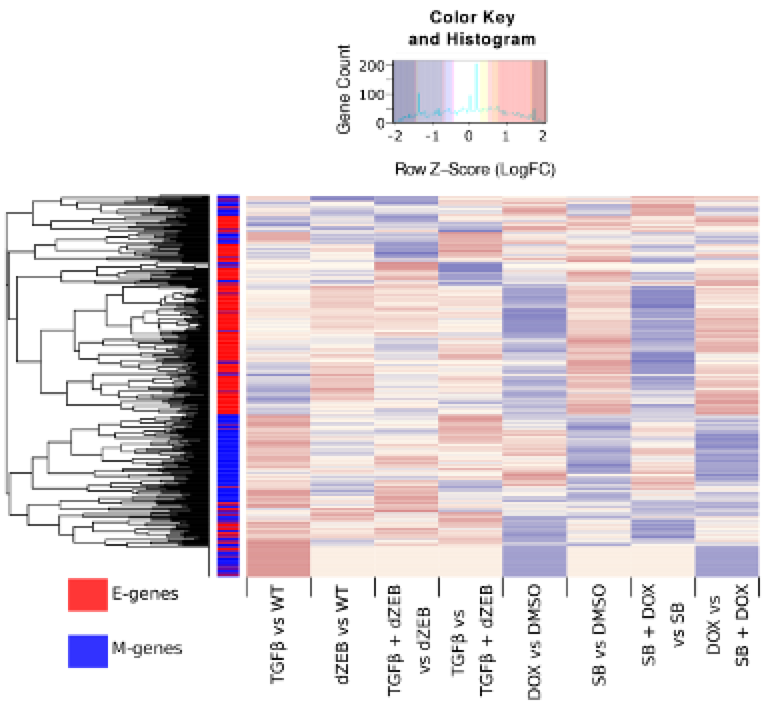

Supplementary Figure 5. **Clustering E- and M-genes according to log fold-change of expression.** Log fold-change of expression of EMT genes in the 8 contrasts conditions (see **Table 2**). Expression values were scaled across genes (rows) to adjust for different average expression between genes. Cells in the heatmap are colored based on the z-score of each expression value in its respective row, indicating whether the gene is expressed relatively higher (yellow) or lower (orange) in that condition than on average. The order of genes was determined by hierarchical clustering without accounting for annotation. E (red) or M (blue) gene annotation is indicated by the color of the left side bar.

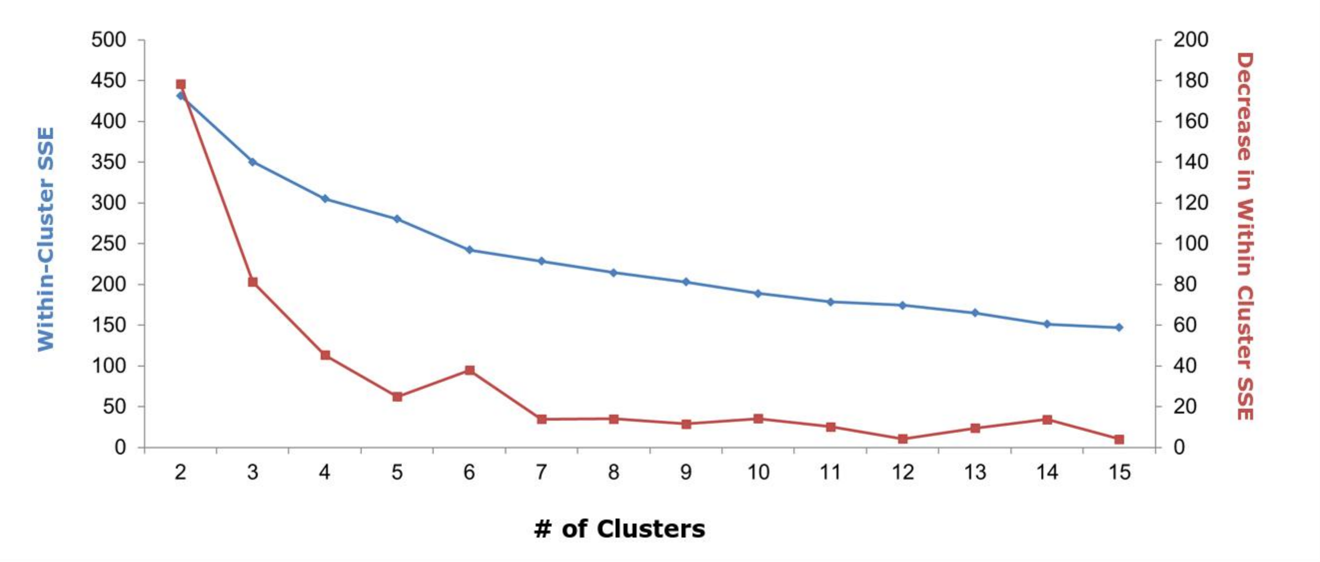

Supplementary Figure 6. **Elbow plot showing the changes of sum of squared errors as the number of clusters increases.** Hierarchical clustering was performed on the average log fold-change of expression of each of the 38 M-gene nodes. Within-Cluster standard squared errors were calculated with different choices of number of clusters for cutting the phylogenetic tree. Blue curve: sum of squared errors. Red curve: changes of Within-Cluster standard squared errors when the number of clusters changes from n-1 to n.

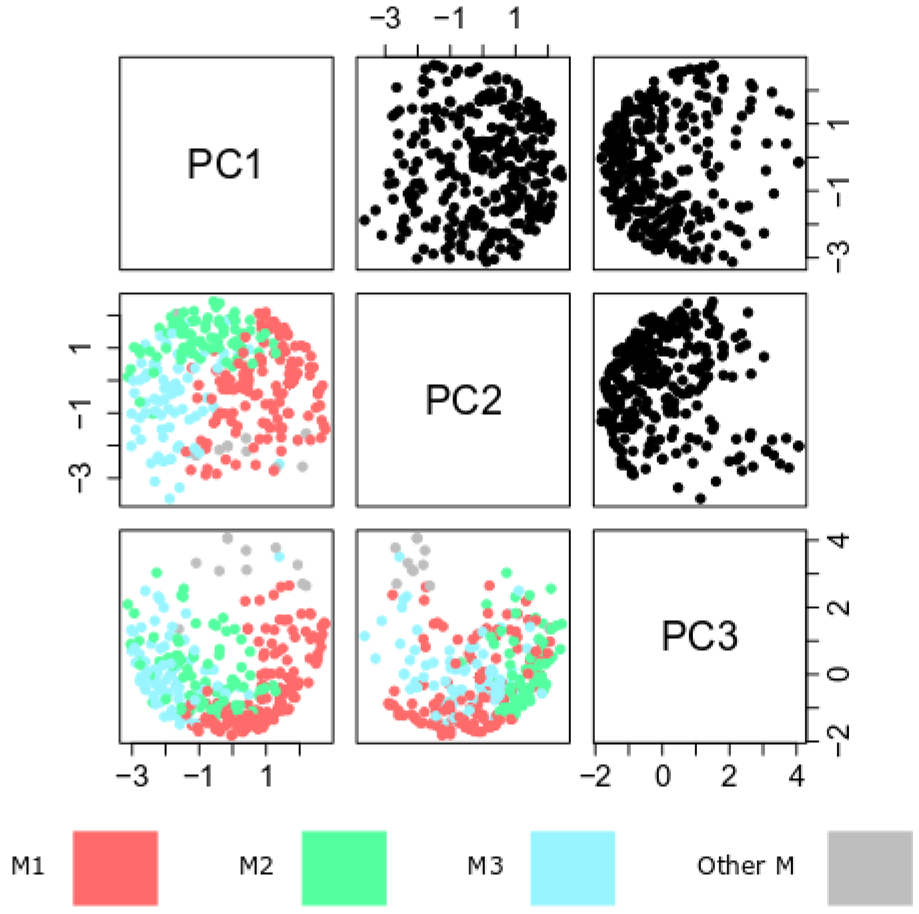

Supplementary Figure 7. Principal Component Analysis of CAGE expression data for M-genes. Lower triangle: M-genes plotted against pairs of the first three principal components of the normalized CAGE expressed data set labeled according to their cluster (M1 = red, M2 = green, M3 = blue, Other M = grey). Upper triangle: the same data points as those in the lower triangle without the cluster labels.

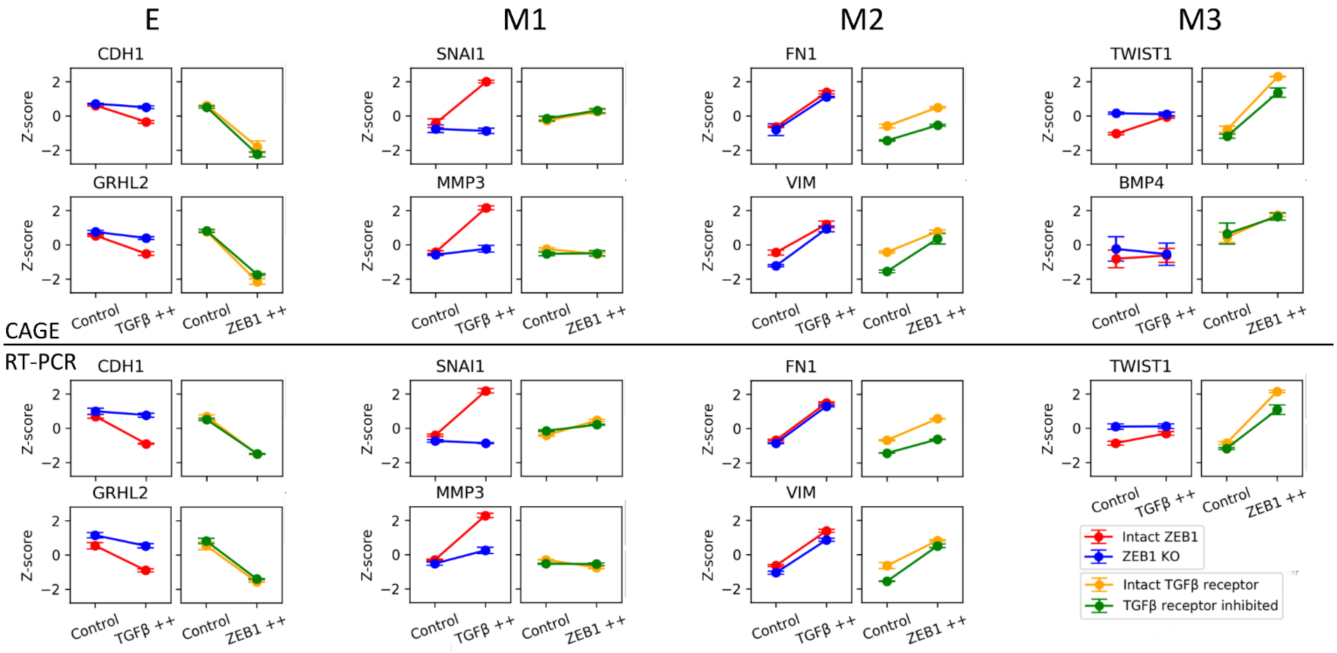

Supplementary Figure 8. Comparison of expression patterns representative genes in four clusters using CAGE and RT-PCR. Expression values of representative genes from each cluster were extracted from the CAGE data set. RT-PCR was performed to verify their expression. 2 replicates were used for each gene at each condition. Error bars indicate the standard error of the means. Z-scores were calculated by centering each of the eight data points to 0 and scale them to unit variance.

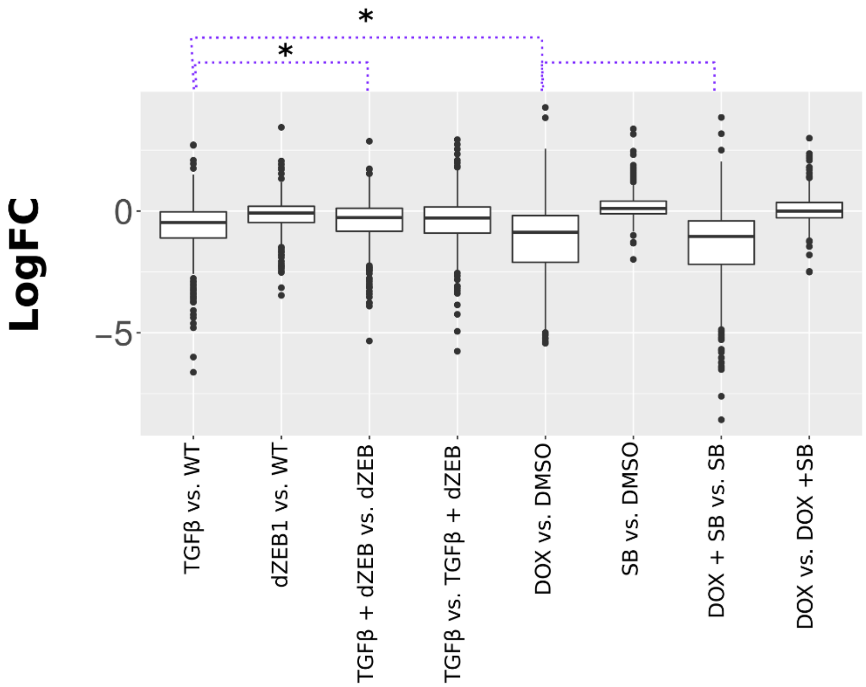

Supplementary Figure 9. Expression of E-gene cluster under TGF-β and ZEB1 regulation. Boxplot of Log-Fold expression of E-genes annotated by SOM clustering in the 8 combination of TGF-β and ZEB1 treatment. The middle region indicates the inter-quartile range of expression while whiskers extend 1.5 times this range on either side. Outliers are indicated by black dots. The purple dotted lines above the plot indicate comparisons between the expression under conditions, specifically, TGF-β vs. WT to DOX vs. DMSO, TGF-β vs. WT to TGF-β + dZEB vs. dZEB and DOX vs. DMSO to DOX + SB vs. SB. A * indicates that distribution of expression is significantly different based on the Mann-Whitney U-test. Note that E-genes have a similar response patter to M3 genes (**Figure 3c)** but the direction of response is reversed (repression in E-genes vs. activation in M3 genes).

Data	Comparison	Δ Log Odds	P-value
TGF-β	M1 vs M2	-0.458	0.161
TGF-β	M1 vs M3	0.342	0.368
TGF-β	M2 vs M3	0.800	**0.053**
Twist	M1 vs M2	-0.259	0.405
Twist	M1 vs M3	-0.158	0.622
Twist	M2 vs M3	0.100	0.782
Snail	M1 vs M2	-0.400	0.186
Snail	M1 vs M3	0.016	0.960
Snail	M2 vs M3	0.416	0.251
Gsc	M1 vs M2	-1.114	**0.0002**
Gsc	M1 vs M3	-0.603	**0.051**
Gsc	M2 vs M3	0.510	0.134
sh-Ecad	M1 vs M2	-0.260	0.411
sh-Ecad	M1 vs M3	-0.003	0.992
sh-Ecad	M2 vs M3	0.256	0.0495

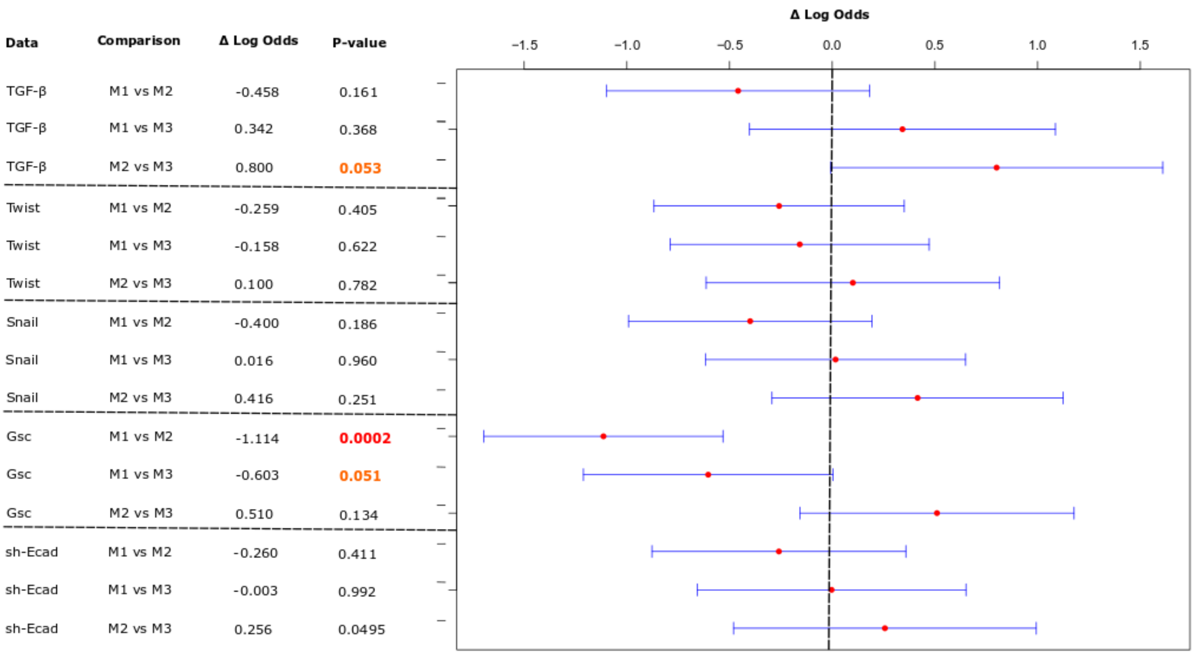

Supplementary Figure 10. Significance and confidence intervals for difference in odds of enrichment between M-gene groups across different regulatory data sets. Left: A table of values showing the significance of enrichment of M1, M2, and M3 genes among genes differentially expressed during the overexpression of TGF-β, Twist, Snail, Gsc or the knockdown of E-cadherin (sh-Ecad). For each comparison between M-gene groups, mean difference in odds is given followed by the two-tailed p-value. Right: 95% confidence intervals of each comparison, where the red dot indicates the average value and blue whiskers indicate the interval range. The dotted line is located at zero which represent no difference in the log of the odds ratio of enrichment between the compared M-gene clusters.

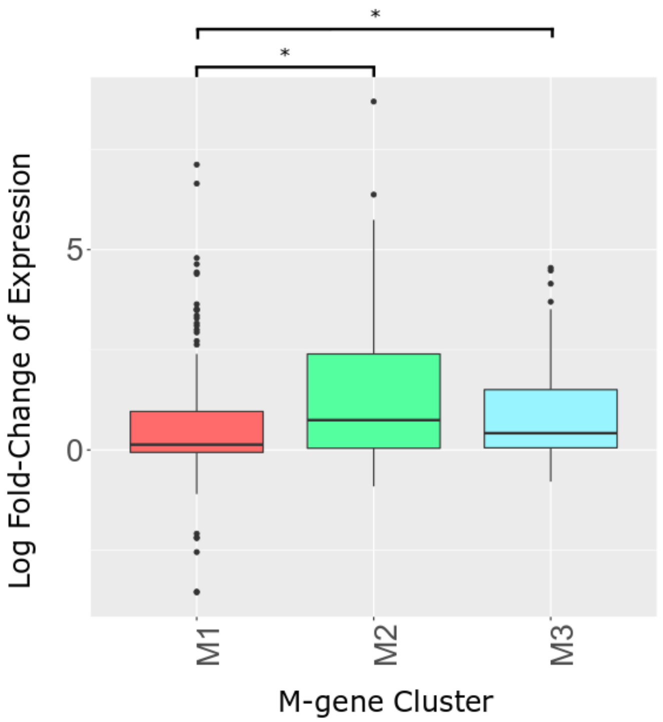

Supplementary Figure 11. Log fold-change of expression of each M-gene cluster in response to GSC overexpression. Boxplot of log fold-change of expression of M-genes cluster in response to the overexpression of Gsc in microarray data from Taube et al. [1]. The colored region (M1 = red, M2 = green, M3 = blue) indicates the inter-quartile range of expression while whiskers extend 1.5 times this range on either side. Outliers are indicated by black dots. The lines above the plot indicate comparisons between the expression under conditions, specifically, M1 vs M2 and M1 and M3. A * indicates that distribution of expression is significantly different based on the Mann-Whitney U-test at an alpha of 0.05.

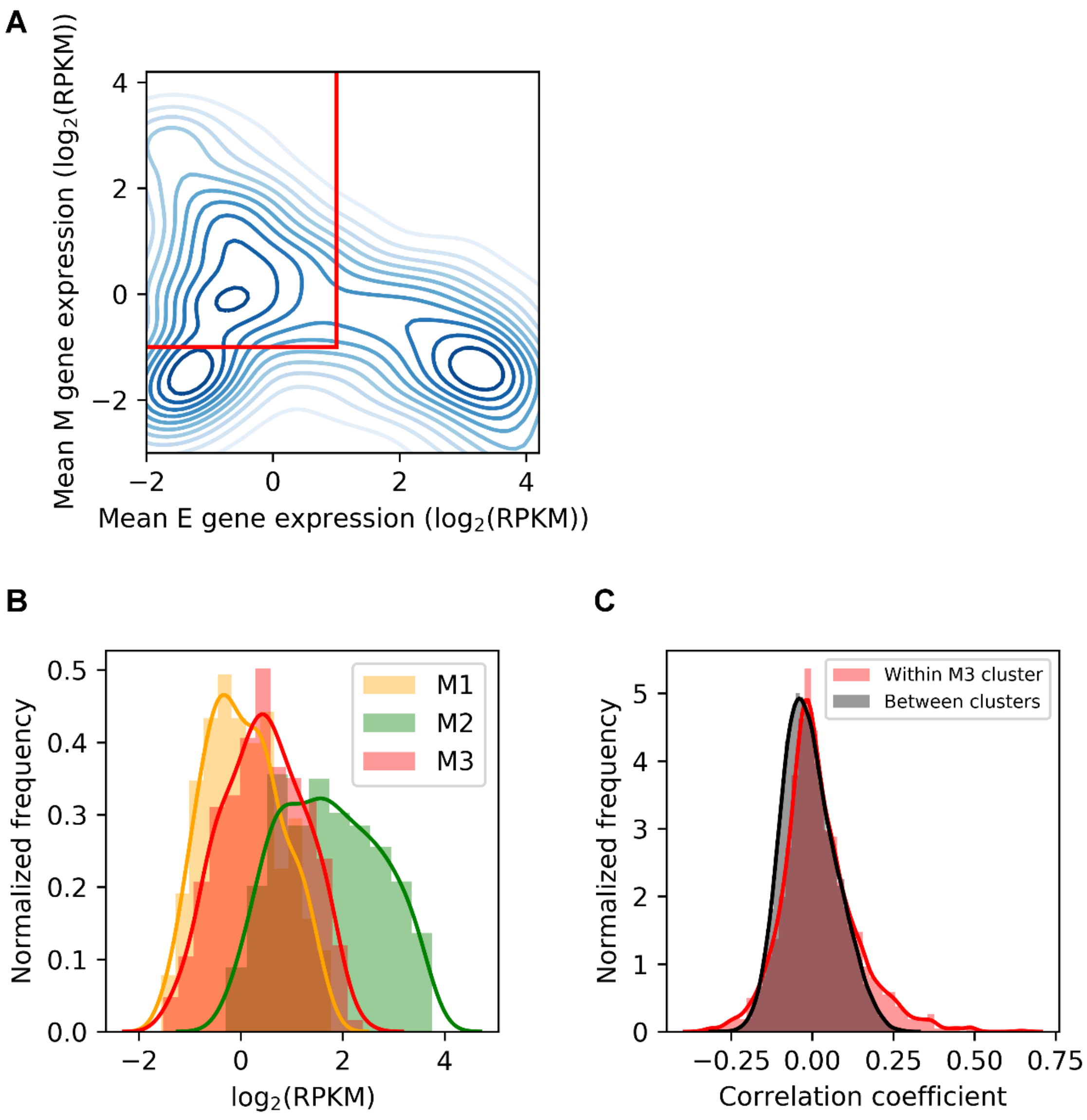

Supplementary Figure 12. Expression of three cluster of M-genes in cancer cell lines. A. Mean expressions of E-genes and M-genes in 1019 cancer cell lines [5]. Red lines indicate the thresholds for selecting mesenchymal-like cancer cells (mean M-gene expression $\log_2$(RPKM) > -1, mean E-gene expression $\log_2$(RPKM) < 1). Contour lines indicate densities of cells. **B.** Mean expressions of M-gene clusters in 416 mesenchymal-like cancer cells. **C.** Pearson correlations between pairs of M3 genes (red), and those between M3 genes and M1/M2 genes (gray). Histograms show the frequencies. Curves show kernel density estimates.

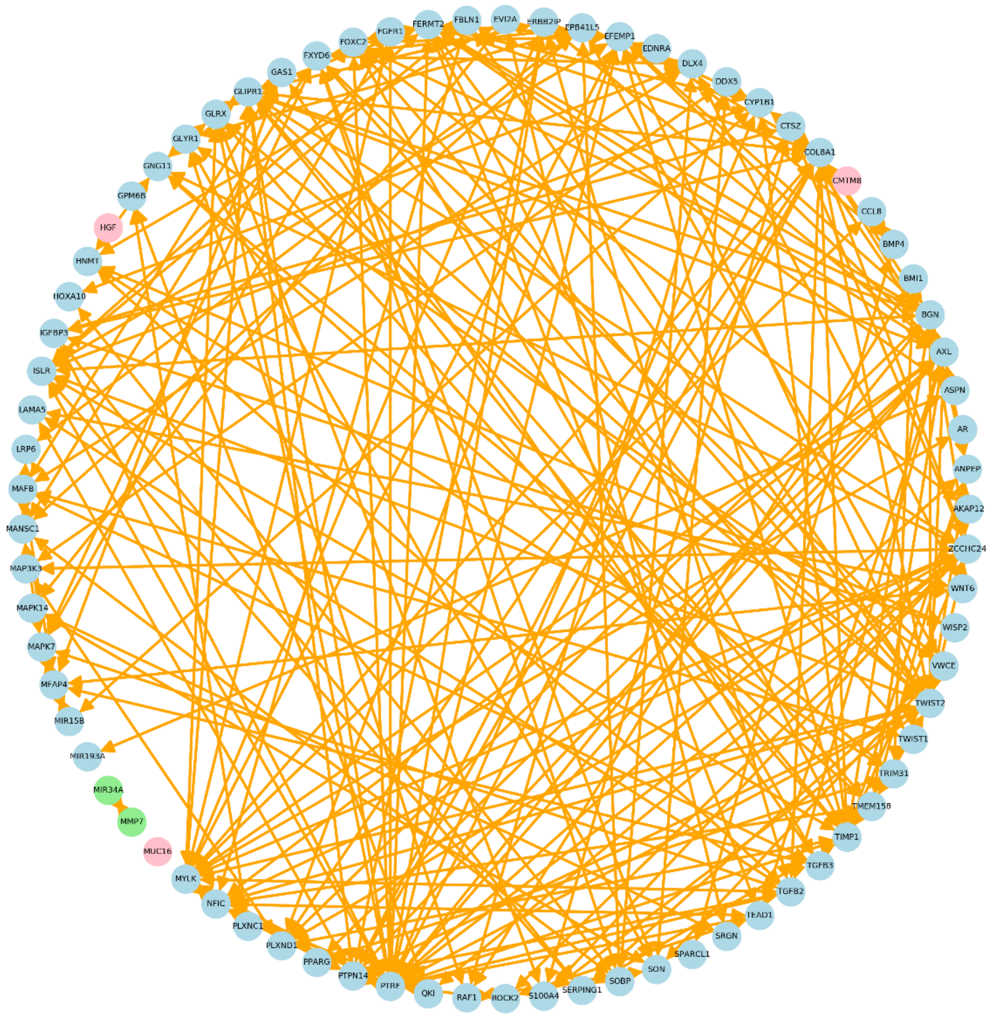

Supplementary Figure 13. Correlation network of M3 genes. Correlation based network of M3 genes obtained from the analysis of gene expression in 416 mesenchymal-like cancer cells (**Supplementary Method 3**). Correlation threshold (r > 0.25) was chosen based on distributions of correlation coefficients of within-cluster correlations and between-cluster correlations (**Supplementary Figure 13C**). The threshold is above the maximal correlation coefficient of the between-cluster correlations. Orange lines denote the association between genes using this threshold. Blue icons show 73 M3 genes forming a large cluster based on these associations. Green and pink icons show 4 M3 genes that are not in the cluster.

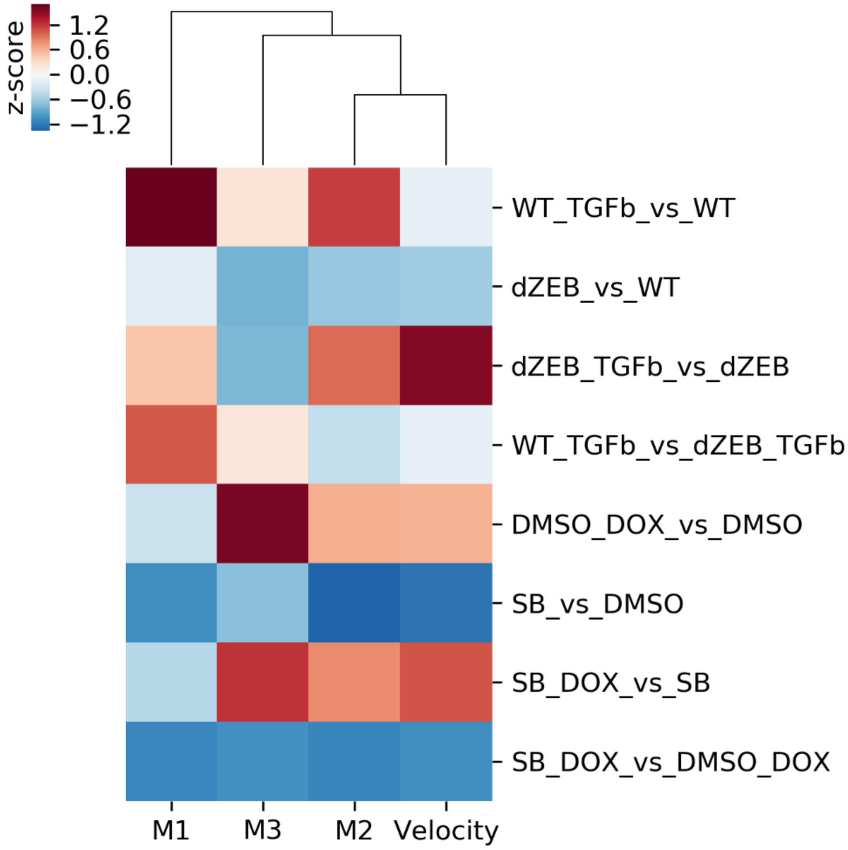

Supplementary Figure 14. Correlation between changes of mean gene expression and velocity of cells under 8 contrast conditions. The fold-changes of mean gene expression of three M-gene clusters under 8 contrast experimental conditions were clustered together with that of the velocity of cells under the same conditions. Column z-scores were computed to generate the heatmap

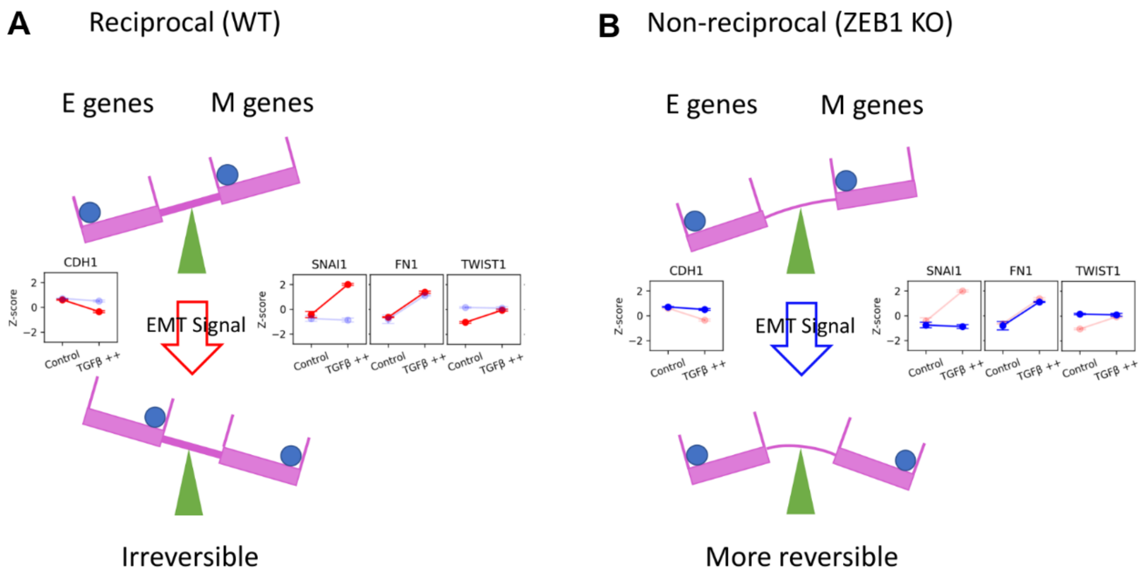

Supplementary Figure 15. Metaphoric illustration of relationship between reciprocity of E- and M-genes and irreversibility of EMT transition. (A) Under normal condition, E- and M-genes are reciprocally regulated and both balls representing the feedback loops involving E- and M-genes move upon the external force followed by the flip of the seesaw, which makes the EMT irreversible **(B)** Under ZEB1 knockout condition, reciprocity of E- and M-genes is compromised and some of the feedback loops are no longer functioning. EMT becomes less irreversible in this scenario.

SUPPLEMENTARY TABLES

Supplementary Table 1. Overlapping EMT gene expression before and after annotating dbEMT genes

Overlap Condition	Class	Original Annotation	Updated Annotation
TGF-β and ZEB1	E	67.1%	55.5%
	M	35.6%	30.1%
TGF-β and TGF-β w/o ZEB1	E	63.3%	46.9%
	M	46.1%	47.2%
ZEB1 and ZEB1 w/o TGF-β	E	93.6%	77.3%
	M	67.5%	60.0%

Supplementary Table 2. List of EMT genes analyzed in this study and their clustering information

Gene	Cluster	Annotation	Source	P_values	LEC_months	HEC_months
ABCC3	E	E	Tan et al., 2014	0.6026	228.85	216.66
ABHD11	E	E	Tan et al., 2014	0.0013	228.85	216.66
ADAM17	E	NA	dbEMT	0.032	56.6	42
ADAP1	E	E	Tan et al., 2014	5.20E-08	39	64
AGER	E	NA	dbEMT	0.0002	42	59
AGR2	E	E	Tan et al., 2014	0.0171	29	40.3
AIM1	E	E	Tan et al., 2014	0.0301	55.43	42
AKR1B10	E	E	Tan et al., 2014	0.3266	228.85	191.21
ALDH3B2	E	E	Tan et al., 2014	0.0357	216.66	191.21
ALOX5	E	E	Tan et al., 2014	1.80E-06	184.04	216.66
AMHR2	E	NA	dbEMT	1.60E-09	38	66
ANK2	E	M	Tan et al., 2014	2.90E-12	35.04	69.7
ANK3	E	E	Tan et al., 2014	1.60E-07	28.96	46.8
ANXA1	E	NA	dbEMT	0.3191	53	45.6
ANXA4	E	E	Tan et al., 2014	0.6625	47.51	48
ANXA9	E	E	Tan et al., 2014	3.60E-05	228.85	216.66
AP1M2	E	E	Tan et al., 2014	0.2305	228.85	216.66
AQP3	E	E	Tan et al., 2014	0.0001	58	41.69
ARAP2	E	E	Tan et al., 2014	0.6035	50.4	46
AREG	E	E	Tan et al., 2014	0.0024	42.3	56.4
ARHGAP8	E	E	Tan et al., 2014	8.90E-07	40.08	62
ARHGDIB	E	E	Tan et al., 2014	5.40E-07	38.4	61
ARHGEF5	E	E	Tan et al., 2014	0.3123	228.85	216.66
ATP2C2	E	E	Tan et al., 2014	0.0659	52.83	44
AURKA	E	NA	dbEMT	1.00E-16	216.66	163.46
AXIN1	E	NA	dbEMT	5.60E-06	191.21	216.66
AZGP1	E	E	Tan et al., 2014	7.10E-06	40.56	61
B3GNT3	E	E	Tan et al., 2014	0.6972	185.16	216.66
BCAS1	E	E	Tan et al., 2014	0.0125	216.66	228.85
BCL2	E	NA	dbEMT	9.90E-11	191.21	216.66
BCL2L1	E	NA	dbEMT	1.90E-05	228.85	216.66
BIK	E	E	Tan et al., 2014	0.4743	48	48
BLNK	E	E	Tan et al., 2014	0.0001	228.85	216.66
BMP7	E	NA	dbEMT	0.0232	228.85	216.66
BOP1	E	NA	dbEMT	0.0002	57.27	41.64
BSPRY	E	E	Tan et al., 2014	6.90E-08	171.43	216.66
C1orf106	E	E	Tan et al., 2014	4.70E-11	228.85	184.04
C1orf116	E	E	Tan et al., 2014	0.0006	216.66	185.16
C1orf54	E	M	Tan et al., 2014	0.5834	216.66	228.85
C4orf19	E	E	Tan et al., 2014	0.126	38	33
CAMK2N1	E	E	Tan et al., 2014	0.0044	216.66	191.21
CAPN1	E	E	Tan et al., 2014	0.2362	228.85	216.66
CAV1	E	M	Tan et al., 2014	0.0117	216.66	228.85
CBLC	E	E	Tan et al., 2014	0.2914	228.85	216.66

CBR1	E	NA	dbEMT	0.0593	53.04	44.4
CD2AP	E	E	Tan et al., 2014	4.20E-06	216.66	191.21
CD46	E	E	Tan et al., 2014	0.0103	55.66	45
CD9	E	E	Tan et al., 2014	0.0101	216.66	228.85
CDH1	E	E	Tan et al., 2014	2.80E-05	59	40.56
CDH3	E	E	Tan et al., 2014	0.0108	216.66	228.85
CDS1	E	E	Tan et al., 2014	0.4644	48	48
CEACAM1	E	E	Tan et al., 2014	0.0119	228.85	191.21
CEACAM5	E	E	Tan et al., 2014	0.691	228.85	216.66
CEACAM6	E	E	Tan et al., 2014	0.0234	228.85	184.04
CLDN1	E	NA	dbEMT	0.3356	39	30.72
CLDN4	E	E	Tan et al., 2014	0.1675	228.85	216.66
CLDN7	E	E	Tan et al., 2014	0.1046	51	46
CLEC2B	E	M	Tan et al., 2014	1.40E-05	28.35	44
CLU	E	NA	dbEMT	1.20E-11	37.2	69
CNKSR1	E	E	Tan et al., 2014	2.20E-15	150.54	216.66
COL14A1	E	M	Tan et al., 2014	1.00E-06	28	46
COMT	E	E	Tan et al., 2014	0.1553	228.85	216.66
CORO2A	E	E	Tan et al., 2014	0.174	31.56	39
CRISPLD2	E	M	Tan et al., 2014	8.90E-05	185.16	216.66
CSF2RB	E	M	Tan et al., 2014	2.20E-09	39	65.04
CSK	E	NA	dbEMT	0.2892	48	48
CSRP2	E	M	Tan et al., 2014	7.60E-07	216.66	228.85
CTNNB1	E	NA	dbEMT	0.038	46	53.16
CTNNBIP1	E	NA	dbEMT	0.0027	43	57.3
CTNND1	E	NA	dbEMT	0.0494	44.22	53.1
CTSH	E	E	Tan et al., 2014	0.0377	43	55.13
CXADR	E	E	Tan et al., 2014	0.0011	42	28.35
CXCL16	E	NA	dbEMT	0.0052	34	38.4
CYP4F3	E	E	Tan et al., 2014	4.60E-05	39.95	60
DAPK1	E	NA	dbEMT	0.1356	48	48
DDR1	E	E	Tan et al., 2014	0.0529	216.66	228.85
DENND2D	E	E	Tan et al., 2014	1.20E-14	46	53.16
DHCR24	E	E	Tan et al., 2014	4.00E-06	43	57.3
DPYSL3	E	M	Tan et al., 2014	0.0031	44.22	53.1
DSC2	E	E	Tan et al., 2014	0.0002	43	55.13
DSG2	E	E	Tan et al., 2014	5.50E-07	42	28.35
DSP	E	E	Tan et al., 2014	0.4936	34	38.4
DTX4	E	E	Tan et al., 2014	0.42	39.95	60
ECT2	E	NA	dbEMT	1.00E-16	53.16	44.22
EGF	E	NA	dbEMT	0.0003	44.17	53.56
EGFR	E	NA	dbEMT	0.0736	171.43	216.66
EGR1	E	NA	dbEMT	2.90E-05	40	60.12
EHF	E	E	Tan et al., 2014	0.3975	228.85	216.66
EIF3I	E	NA	dbEMT	0.0148	43	28.8
EIF5A2	E	NA	dbEMT	0.0127	61.2	38.7
ELF3	E	E	Tan et al., 2014	0.0773	228.85	216.66
ELL3	E	NA	dbEMT	1.50E-05	48	48
ELMO3	E	E	Tan et al., 2014	0.3213	216.66	171.43
EPAS1	E	NA	dbEMT	0.0469	191.21	228.85

EPB41L4B	E	E	Tan et al., 2014	0.0232	35	37
EPCAM	E	E	Tan et al., 2014	1.00E-16	185.16	216.66
EPHA1	E	E	Tan et al., 2014	7.80E-10	38.4	32.4
EPN3	E	E	Tan et al., 2014	0.0167	191.21	228.85
EPS8L1	E	E	Tan et al., 2014	0.0003	31.56	40
EPS8L2	E	E	Tan et al., 2014	0.0389	34	38.4
ERBB2	E	E	Tan et al., 2014	0.1804	62	40
ERBB3	E	E	Tan et al., 2014	0.0011	228.85	216.66
ERMP1	E	E	Tan et al., 2014	0.6614	44.22	55
ESR1	E	NA	dbEMT	2.50E-13	30.72	40.44
ESRP1	E	E	Tan et al., 2014	4.40E-12	77.23	33.63
ESRP2	E	E	Tan et al., 2014	0.0006	191.21	216.66
ETV1	E	M	Tan et al., 2014	8.10E-09	216.66	185.16
ETV4	E	NA	dbEMT	0.8505	228.85	216.66
EVPL	E	E	Tan et al., 2014	0.1597	228.85	216.66
EXPH5	E	E	Tan et al., 2014	0.0065	228.85	216.66
EZH2	E	NA	dbEMT	4.70E-13	27	43.93
EZR	E	E	Tan et al., 2014	0.0081	36	36.9
F11R	E	E	Tan et al., 2014	0.6525	184.04	216.66
FA2H	E	E	Tan et al., 2014	0.0091	51.36	27.4
FGF1	E	NA	dbEMT	4.70E-05	57	41.95
FGFR3	E	E	Tan et al., 2014	0.0077	228.85	216.66
FHL2	E	NA	dbEMT	0.0004	228.85	216.66
FLI1	E	M	Tan et al., 2014	0.5315	228.85	216.66
FLT1	E	NA	dbEMT	0.5276	228.85	216.66
FOSL1	E	NA	dbEMT	0.6232	74	34.8
FOXA1	E	E	Tan et al., 2014	3.10E-08	55.99	44
FOXM1	E	NA	dbEMT	1.00E-16	50	46.8
FOXQ1	E	NA	dbEMT	0.657	55	43
FSCN1	E	NA	dbEMT	0.0808	42	59
FUT2	E	E	Tan et al., 2014	2.40E-05	42	55.2
FUT3	E	E	Tan et al., 2014	0.1216	41.95	60
FXYD3	E	E	Tan et al., 2014	6.30E-06	228.85	191.21
GALE	E	E	Tan et al., 2014	0.022	37.64	34.82
GALNT3	E	E	Tan et al., 2014	2.20E-12	191.21	216.66
GALNT7	E	E	Tan et al., 2014	0.0948	191.21	216.66
GATA3	E	NA	dbEMT	1.00E-05	37.64	60
GDF15	E	E	Tan et al., 2014	0.5517	228.85	216.66
GEMIN2	E	NA	dbEMT	6.00E-08	216.66	185.16
GIMAP6	E	M	Tan et al., 2014	0.0004	216.66	228.85
GMDS	E	E	Tan et al., 2014	0.3653	52.54	41.69
GMNN	E	NA	dbEMT	1.00E-16	216.66	184.04
GPR56	E	E	Tan et al., 2014	9.80E-14	228.85	171.43
GPRC5A	E	E	Tan et al., 2014	0.0007	216.66	191.21
GPX2	E	E	Tan et al., 2014	6.50E-05	228.85	216.66
GRB7	E	E	Tan et al., 2014	0.0069	228.85	216.66
GRHL1	E	E	Tan et al., 2014	0.882	37	34.8
GRHL2	E	E	Tan et al., 2014	0.0001	216.66	184.04
GRHL3	E	E	Tan et al., 2014	0.9686	34.8	37
GSK3A	E	NA	dbEMT	2.00E-08	191.21	216.66

GSK3B	E	NA	dbEMT	0.3888	36.44	35
HBEGF	E	NA	dbEMT	9.00E-06	184.04	216.66
HDGF	E	NA	dbEMT	6.40E-07	228.85	216.66
HDHD3	E	E	Tan et al., 2014	0.0161	228.85	216.66
HES1	E	E	Tan et al., 2014	0.0128	184.04	216.66
HMGB1	E	NA	dbEMT	0.8187	36.44	36
HPGD	E	E	Tan et al., 2014	0.0009	43	57.6
HPSE	E	NA	dbEMT	1.70E-07	28.8	46.8
HRAS	E	NA	dbEMT	0.0409	53.28	44.22
HS6ST2	E	NA	dbEMT	0.5385	36.96	35
HSP90AA1	E	NA	dbEMT	5.90E-15	216.66	184.04
HSPA4	E	NA	dbEMT	4.30E-07	216.66	185.16
ICA1	E	E	Tan et al., 2014	7.30E-11	228.85	216.66
ID1	E	NA	dbEMT	0.0002	216.66	228.85
ID2	E	NA	dbEMT	0.0757	55.13	43
IDH1	E	NA	dbEMT	2.10E-06	61.05	39
IDH2	E	NA	dbEMT	7.80E-14	216.66	184.04
IFFO1	E	M	Tan et al., 2014	2.50E-10	37.8	65.04
IL18	E	NA	dbEMT	4.90E-05	216.66	228.85
IL1B	E	NA	dbEMT	1.40E-11	171.43	216.66
IL1RN	E	E	Tan et al., 2014	0.0028	44	54.77
IL20RA	E	E	Tan et al., 2014	2.50E-12	171.43	216.66
IL6R	E	NA	dbEMT	6.50E-05	30	43
ILK	E	NA	dbEMT	8.20E-05	228.85	216.66
IRF6	E	E	Tan et al., 2014	0.6955	228.85	216.66
ITGA6	E	NA	dbEMT	0.654	51	46.68
ITGB3	E	NA	dbEMT	0.4851	228.85	191.21
ITGB4	E	E	Tan et al., 2014	0.0022	228.85	216.66
JAG1	E	NA	dbEMT	0.636	228.85	216.66
JAG2	E	NA	dbEMT	2.50E-05	216.66	228.85
JUP	E	E	Tan et al., 2014	0.2519	228.85	216.66
KCNK1	E	E	Tan et al., 2014	4.80E-08	228.85	216.66
KL	E	NA	dbEMT	2.70E-11	37	66
KLF5	E	E	Tan et al., 2014	8.70E-06	228.85	216.66
KLF6	E	NA	dbEMT	0.7772	38	31.51
KLF8	E	M	Tan et al., 2014	0.0079	58	41.04
KLK6	E	E	Tan et al., 2014	0.0628	228.85	185.16
KRAS	E	NA	dbEMT	0.0003	191.21	228.85
KRT15	E	E	Tan et al., 2014	0.0044	45	55.9
KRT18	E	E	Tan et al., 2014	0.7277	46.8	50.3
KRT19	E	E	Tan et al., 2014	0.1144	48	48.16
KRT7	E	E	Tan et al., 2014	0.0029	228.85	216.66
L1CAM	E	NA	dbEMT	0.6474	191.21	216.66
LAD1	E	E	Tan et al., 2014	0.0237	216.66	184.04
LAMA1	E	NA	dbEMT	0.5511	35	37.2
LATS1	E	NA	dbEMT	1.60E-06	28.09	44
LCN2	E	E	Tan et al., 2014	0.0772	228.85	216.66
LETMD1	E	NA	dbEMT	1.10E-16	35	75
LIMA1	E	NA	dbEMT	1.10E-06	39	61.92
LLGL2	E	E	Tan et al., 2014	0.6439	51	47

LOXL2	E	M	Tan et al., 2014	0.0004	228.85	216.66
LOXL3	E	NA	dbEMT	0.0497	33	39
LRRC1	E	E	Tan et al., 2014	4.80E-05	216.66	191.21
LSR	E	E	Tan et al., 2014	2.90E-11	69.6	35.98
LY75	E	E	Tan et al., 2014	0.0002	191.21	228.85
LY96	E	M	Tan et al., 2014	0.1804	216.66	191.21
LYPD3	E	NA	dbEMT	0.0001	191.21	228.85
MALL	E	E	Tan et al., 2014	0.7841	228.85	216.66
MAP2K1	E	NA	dbEMT	0.8283	50	47
MAP7	E	E	Tan et al., 2014	0.002	216.66	191.21
MAPK13	E	E	Tan et al., 2014	0.0032	58	40.8
MAPK3	E	NA	dbEMT	3.80E-05	228.85	216.66
MAPK8	E	NA	dbEMT	1.00E-16	95	171.43
MARVELD3	E	NA	dbEMT	0.0228	36.96	35
MET	E	NA	dbEMT	0.3528	47.4	49.45
MGAT3	E	NA	dbEMT	0.085	45.37	53.36
MGST2	E	E	Tan et al., 2014	0.0001	216.66	228.85
MIR200C	E	NA	dbEMT	n/a	n/a	n/a
MIR221	E	NA	dbEMT	n/a	n/a	n/a
MIR222	E	NA	dbEMT	n/a	n/a	n/a
MIR23A	E	NA	dbEMT	n/a	n/a	n/a
MIR30A	E	NA	dbEMT	n/a	n/a	n/a
MIR33A	E	NA	dbEMT	n/a	n/a	n/a
MKL2	E	NA	dbEMT	0.0032	35.04	68.4
MPZL2	E	E	Tan et al., 2014	3.80E-05	39	30.42
MST1R	E	E	Tan et al., 2014	1.00E-16	191.21	216.66
MSX2	E	NA	dbEMT	0.0228	41.72	57
MTA1	E	NA	dbEMT	0.3528	191.21	216.66
MTA3	E	NA	dbEMT	0.085	27	49
MTOR	E	NA	dbEMT	0.0001	42.96	57
MTUS1	E	E	Tan et al., 2014	2.20E-11	39	61
MUC1	E	E	Tan et al., 2014	0.7243	228.85	216.66
MUC2	E	NA	dbEMT	5.10E-06	216.66	228.85
MYC	E	NA	dbEMT	0.0003	228.85	216.66
MYH10	E	M	Tan et al., 2014	2.70E-06	228.85	191.21
MYO1D	E	E	Tan et al., 2014	5.30E-09	191.21	216.66
MYO5C	E	E	Tan et al., 2014	3.90E-05	185.16	216.66
MYO6	E	E	Tan et al., 2014	5.00E-07	191.21	216.66
NDRG1	E	NA	dbEMT	0.0019	216.66	184.04
NFKB1	E	NA	dbEMT	1.20E-08	191.21	216.66
NOTCH1	E	NA	dbEMT	0.0493	191.21	216.66
NOTCH2	E	NA	dbEMT	0.2878	44.17	55
NQO1	E	E	Tan et al., 2014	0.207	216.66	185.16
NRP2	E	NA	dbEMT	1.30E-14	37	64.73
NUMB	E	NA	dbEMT	0.0047	54	43
OAS1	E	E	Tan et al., 2014	2.20E-09	216.66	191.21
OCLN	E	E	Tan et al., 2014	3.30E-16	25.2	50.4
OR7E14P	E	E	Tan et al., 2014	0.9091	29	42
OVOL1	E	E	Tan et al., 2014	0.0049	184.04	216.66
OVOL2	E	E	Tan et al., 2014	4.70E-06	31.51	43

PAG1	E	NA	dbEMT	9.00E-06	228.85	216.66
PAK1	E	NA	dbEMT	0.4208	42	57
PARD3	E	NA	dbEMT	0.5676	228.85	216.66
PARP1	E	NA	dbEMT	6.60E-09	216.66	184.04
PCMT1	E	NA	dbEMT	0.0009	51	46.8
PDGFB	E	NA	dbEMT	5.20E-06	52.9	45
PDPN	E	NA	dbEMT	1.00E-04	38	33
PERP	E	E	Tan et al., 2014	2.10E-09	35	36.9
PHLDA1	E	NA	dbEMT	0.0107	228.85	173.2
PHLDA2	E	E	Tan et al., 2014	0.0569	216.66	185.16
PIN1	E	NA	dbEMT	2.30E-05	228.85	216.66
PKP3	E	E	Tan et al., 2014	0.4644	228.85	216.66
PLAUR	E	NA	dbEMT	0.1181	185.16	216.66
PLLP	E	E	Tan et al., 2014	0.0983	48.96	48
PLS1	E	E	Tan et al., 2014	0.4374	33	38
POF1B	E	E	Tan et al., 2014	4.00E-10	228.85	216.66
PPAP2C	E	E	Tan et al., 2014	0.0007	228.85	216.66
PPFIBP2	E	E	Tan et al., 2014	0.1244	228.85	216.66
PPL	E	E	Tan et al., 2014	0.7174	216.66	228.85
PRDX1	E	NA	dbEMT	7.10E-13	32	39
PRKCE	E	NA	dbEMT	0.5707	52.32	44.22
PROM1	E	NA	dbEMT	0.1483	228.85	216.66
PRR15L	E	E	Tan et al., 2014	n/a	n/a	n/a
PRSS8	E	E	Tan et al., 2014	0.1483	52.83	45.84
PSCA	E	E	Tan et al., 2014	3.50E-06	185.16	216.66
PTHLH	E	NA	dbEMT	1.10E-06	216.66	191.21
PTK2	E	NA	dbEMT	0.5338	29	45.6
PTK6	E	E	Tan et al., 2014	0.375	216.66	228.85
PTPRC	E	M	Tan et al., 2014	0.1231	228.85	216.66
PTPRF	E	E	Tan et al., 2014	0.0629	43.7	57.6
PTPRZ1	E	NA	dbEMT	3.60E-05	191.21	216.66
PYCARD	E	E	Tan et al., 2014	0.0006	28.96	42
RAB11FIP1	E	E	Tan et al., 2014	3.30E-06	44	55.13
RAB20	E	E	Tan et al., 2014	0.0004	228.85	185.16
RAB25	E	E	Tan et al., 2014	0.0198	164.3	228.85
RABGAP1L	E	E	Tan et al., 2014	0.0005	228.85	216.66
RAPGEFL1	E	E	Tan et al., 2014	1.10E-05	51.36	46.49
RBM47	E	E	Tan et al., 2014	0.044	228.85	216.66
RGS3	E	NA	dbEMT	0.0006	216.66	228.85
RHOA	E	NA	dbEMT	0.5855	49	47
RHOD	E	E	Tan et al., 2014	3.70E-08	48	48.16
RNF128	E	E	Tan et al., 2014	2.90E-06	216.66	161.29
S100A14	E	E	Tan et al., 2014	0.4268	38	32
S100P	E	E	Tan et al., 2014	0.5239	191.21	216.66
SCEL	E	E	Tan et al., 2014	0.4037	228.85	216.66
SCNN1A	E	E	Tan et al., 2014	0.6697	57	42
SCRIB	E	NA	dbEMT	0.374	44.5	52.54
SDC1	E	E	Tan et al., 2014	2.60E-13	39	31.18
SDC4	E	E	Tan et al., 2014	0.4125	228.85	185.16
SEMA7A	E	NA	dbEMT	8.20E-09	45.6	53.36

SERINC5	E	E	Tan et al., 2014	n/a	n/a	n/a
SFN	E	E	Tan et al., 2014	0.0329	52.83	45.84
SFRP1	E	M	Tan et al., 2014	0.0353	185.16	216.66
SFTPC	E	NA	dbEMT	1.70E-14	216.66	191.21
SH2D3A	E	E	Tan et al., 2014	4.90E-07	29	45.6
SH3YL1	E	E	Tan et al., 2014	0.0022	216.66	228.85
SHANK2	E	E	Tan et al., 2014	0.6314	228.85	216.66
SIM2	E	NA	dbEMT	1.30E-05	43.7	57.6
SLC16A5	E	E	Tan et al., 2014	4.00E-05	191.21	216.66
SLC22A18	E	E	Tan et al., 2014	0.0991	28.96	42
SLC2A3	E	M	Tan et al., 2014	0.616	44	55.13
SLC35A3	E	E	Tan et al., 2014	0.0562	228.85	185.16
SLC37A1	E	E	Tan et al., 2014	0.1087	164.3	228.85
SLC39A6	E	NA	dbEMT	9.90E-08	228.85	216.66
SLC44A4	E	E	Tan et al., 2014	4.00E-09	51.36	46.49
SLC9A3R1	E	E	Tan et al., 2014	0.0028	228.85	216.66
SLIT2	E	M	Tan et al., 2014	1.10E-15	216.66	228.85
SLPI	E	E	Tan et al., 2014	6.00E-08	49	47
SMAD2	E	NA	dbEMT	0.5183	48	48.16
SMAD3	E	NA	dbEMT	0.0069	216.66	161.29
SMAD9	E	NA	dbEMT	2.20E-12	38	32
SNW1	E	NA	dbEMT	0.0002	191.21	216.66
SORD	E	E	Tan et al., 2014	0.0029	228.85	216.66
SORL1	E	E	Tan et al., 2014	8.50E-08	57	42
SOX9	E	NA	dbEMT	0.0369	44.5	52.54
SP1	E	NA	dbEMT	5.20E-08	39	31.18
SPAG1	E	E	Tan et al., 2014	0.471	228.85	185.16
SPDEF	E	E	Tan et al., 2014	3.40E-07	185.16	216.66
SPINT1	E	E	Tan et al., 2014	0.7666	58.52	40.37
SPINT2	E	E	Tan et al., 2014	7.00E-08	228.85	216.66
SPP1	E	NA	dbEMT	4.90E-11	27	45.6
SPRR2A	E	NA	dbEMT	n/a	n/a	n/a
SSH3	E	E	Tan et al., 2014	0.0329	216.66	228.85
ST14	E	E	Tan et al., 2014	0.0353	44	54
STAP2	E	E	Tan et al., 2014	1.70E-14	191.21	216.66
STK11	E	NA	dbEMT	4.90E-07	44.5	54
STYK1	E	E	Tan et al., 2014	0.0022	32	39
SYNGR2	E	E	Tan et al., 2014	0.6314	27.3	46
TACSTD2	E	E	Tan et al., 2014	1.30E-05	228.85	216.66
TBX2	E	NA	dbEMT	4.00E-05	173.2	216.66
TBX20	E	NA	dbEMT	0.0991	25.08	58.15
TCF21	E	NA	dbEMT	0.616	191.21	216.66
TCF7	E	NA	dbEMT	0.0562	51	46
TDGF1	E	NA	dbEMT	0.1087	228.85	191.21
TFCP2	E	NA	dbEMT	9.90E-08	27	57
TFF1	E	E	Tan et al., 2014	4.00E-09	31	40.44
TGFA	E	E	Tan et al., 2014	0.0028	44.4	54.77
TGFB1	E	NA	dbEMT	1.10E-15	228.85	216.66
TGFBR3	E	NA	dbEMT	6.00E-08	228.85	216.66
THBD	E	NA	dbEMT	0.5183	228.85	216.66

TIAM1	E	NA	dbEMT	0.0069	45	52.3
TJP2	E	E	Tan et al., 2014	2.20E-12	228.85	216.66
TJP3	E	E	Tan et al., 2014	0.0002	216.66	191.21
TMC5	E	E	Tan et al., 2014	0.0029	184.04	216.66
TMC6	E	E	Tan et al., 2014	8.50E-08	37.25	69.6
TMEM30B	E	E	Tan et al., 2014	0.0369	228.85	216.66
TMPRSS4	E	E	Tan et al., 2014	5.20E-08	216.66	228.85
TNF	E	NA	dbEMT	0.471	37.2	72.2
TNFSF13	E	E	Tan et al., 2014	3.40E-07	31	39
TOB1	E	E	Tan et al., 2014	0.7666	34	37.2
TOM1L1	E	E	Tan et al., 2014	7.00E-08	228.85	216.66
TP63	E	NA	dbEMT	4.90E-11	228.85	216.66
TP73	E	NA	dbEMT	0.4266	60	43
TPD52	E	E	Tan et al., 2014	0.5736	216.66	191.21
TRPM4	E	E	Tan et al., 2014	1.00E-12	171.43	216.66
TSPAN1	E	E	Tan et al., 2014	0.0289	228.85	185.16
TSPAN13	E	E	Tan et al., 2014	0.9798	228.85	216.66
TSPAN15	E	E	Tan et al., 2014	0.6097	36	72
TTC39A	E	E	Tan et al., 2014	0.3555	51.36	45.27
TUBB6	E	M	Tan et al., 2014	1.90E-05	228.85	216.66
TUFT1	E	E	Tan et al., 2014	0.0042	216.66	185.16
TXNIP	E	NA	dbEMT	1.30E-13	171.43	216.66
UCHL1	E	M	Tan et al., 2014	1.50E-05	228.85	216.66
UGT1A1	E	E	Tan et al., 2014	0.5665	66	37
VAMP8	E	E	Tan et al., 2014	1.00E-13	216.66	191.21
VAV3	E	E	Tan et al., 2014	0.4425	43	58
VDR	E	NA	dbEMT	5.40E-05	216.66	228.85
VEGFA	E	NA	dbEMT	0.0183	44.5	56.6
VGLL1	E	E	Tan et al., 2014	2.80E-12	58.65	41
VSNL1	E	NA	dbEMT	0.5664	163.46	216.66
VTN	E	NA	dbEMT	1.80E-07	35	37
WISP3	E	NA	dbEMT	3.10E-06	216.66	171.43
WT1	E	NA	dbEMT	0.0057	76.02	35.09
XBP1	E	E	Tan et al., 2014	0.0149	50	47
YAP1	E	NA	dbEMT	0.017	57.6	27
YBX1	E	NA	dbEMT	0.0004	216.66	228.85
YWHAZ	E	NA	dbEMT	1.00E-15	191.21	216.66
YY1	E	NA	dbEMT	0.3429	60	40.44
ZBTB33	E	NA	dbEMT	1.00E-16	184.04	216.66
ZFYVE9	E	NA	dbEMT	6.70E-16	216.66	191.21
ZNF165	E	E	Tan et al., 2014	0.1806	50	46.32
ZNF217	E	NA	dbEMT	3.20E-12	184.04	216.66
ZYX	E	NA	dbEMT	1.90E-07	228.85	191.21
AKAP2	M1	M	Tan et al., 2014	0.001	36.9	35.09
AKT1	M1	NA	dbEMT	6.20E-05	27	48
AKT3	M1	M	Tan et al., 2014	1.40E-10	216.66	228.85
ANGPTL2	M1	M	Tan et al., 2014	0.0003	228.85	216.66
AP1S2	M1	M	Tan et al., 2014	0.7127	27	49.2
ARHGAP32	M1	E	Tan et al., 2014	2.40E-05	216.66	228.85
ATM	M1	NA	dbEMT	0.3896	228.85	216.66

ATP1B1	M1	E	Tan et al., 2014	0.9761	57.3	40.56
AXIN2	M1	NA	dbEMT	2.10E-07	228.85	185.16
BIRC2	M1	NA	dbEMT	0.0062	228.85	216.66
BMP2	M1	NA	dbEMT	1.00E-07	31	47.28
CALD1	M1	M	Tan et al., 2014	5.50E-08	185.16	216.66
CAMK1D	M1	NA	dbEMT	0.5616	37	34.8
CCL2	M1	M	Tan et al., 2014	0.0199	40	60.6
CD163	M1	M	Tan et al., 2014	0.0056	40.44	60
CD274	M1	NA	dbEMT	0.5148	216.66	228.85
CD44	M1	NA	dbEMT	0.8939	216.66	191.21
CDH11	M1	M	Tan et al., 2014	7.20E-08	61.64	40
CDK14	M1	M	Tan et al., 2014	n/a	n/a	n/a
CDX2	M1	NA	dbEMT	3.90E-09	61	39
CEACAM7	M1	E	Tan et al., 2014	0.5302	228.85	216.66
CHN1	M1	M	Tan et al., 2014	6.30E-05	228.85	216.66
CHRDL1	M1	M	Tan et al., 2014	6.50E-06	228.85	216.66
CLDN3	M1	E	Tan et al., 2014	0.0517	184.04	216.66
CLIC4	M1	M	Tan et al., 2014	2.40E-08	216.66	228.85
COL15A1	M1	M	Tan et al., 2014	4.60E-06	47.04	50.86
COL6A1	M1	M	Tan et al., 2014	0.0004	42	56.58
COL6A2	M1	M	Tan et al., 2014	0.0254	45	52.9
COL8A2	M1	NA	dbEMT	0.0002	216.66	228.85
COLEC12	M1	M	Tan et al., 2014	0.0038	191.21	216.66
CRYAB	M1	M	Tan et al., 2014	0.0013	46.68	52.2
CTSK	M1	M	Tan et al., 2014	0.0021	228.85	216.66
CUX1	M1	NA	dbEMT	0.3705	48.99	47.88
CXCL12	M1	M	Tan et al., 2014	0.003	47.08	48
CXCL13	M1	M	Tan et al., 2014	0.1049	36	36.04
CXCL5	M1	NA	dbEMT	6.10E-09	38	71
CXCR4	M1	M	Tan et al., 2014	0.1052	216.66	228.85
CYB561	M1	E	Tan et al., 2014	0.1408	55.99	43.56
CYR61	M1	NA	dbEMT	0.0028	40.37	58.65
DDR2	M1	M	Tan et al., 2014	0.2749	35.09	36.44
DENND5A	M1	M	Tan et al., 2014	0.0474	228.85	216.66
DPT	M1	M	Tan et al., 2014	0.5559	216.66	228.85
DSE	M1	M	Tan et al., 2014	1.30E-13	216.66	228.85
ECM2	M1	M	Tan et al., 2014	2.80E-05	228.85	216.66
EDN1	M1	NA	dbEMT	0.0269	41	59
EFEMP2	M1	M	Tan et al., 2014	1.10E-05	37	68.48
ELF5	M1	E	Tan et al., 2014	0.0274	47	48.16
ENPP2	M1	M	Tan et al., 2014	0.0094	228.85	216.66
EPO	M1	NA	dbEMT	0.1522	37	64.73
F13A1	M1	M	Tan et al., 2014	5.40E-08	228.85	191.21
FAM174B	M1	E	Tan et al., 2014	0.0414	184.04	216.66
FAP	M1	M	Tan et al., 2014	1.50E-05	30.03	44
FBLN5	M1	NA	dbEMT	9.00E-11	216.66	191.21
FBP1	M1	E	Tan et al., 2014	0.5099	37.8	64
FGF2	M1	NA	dbEMT	9.10E-06	185.16	216.66
FGFR2	M1	NA	dbEMT	1.80E-07	191.21	216.66
FGL2	M1	M	Tan et al., 2014	9.50E-06	184.04	216.66

FHL1	M1	M	Tan et al., 2014	5.70E-07	41.04	60
FLRT2	M1	M	Tan et al., 2014	4.90E-06	185.16	216.66
FOXC1	M1	NA	dbEMT	0.1473	216.66	228.85
FSCN2	M1	NA	dbEMT	2.40E-08	39	31.8
GAB2	M1	NA	dbEMT	0.0281	66	38
GEM	M1	M	Tan et al., 2014	0.0195	228.85	216.66
GFPT2	M1	M	Tan et al., 2014	1.90E-07	35	37.52
GIMAP4	M1	M	Tan et al., 2014	6.00E-05	43.2	58.15
GLIPR2	M1	NA	dbEMT	3.90E-05	228.85	216.66
GREM1	M1	M	Tan et al., 2014	4.20E-05	216.66	228.85
GRIN1	M1	NA	dbEMT	0.4171	228.85	216.66
GSC	M1	M	Tan et al., 2014	6.90E-07	31.56	40.53
GSN	M1	NA	dbEMT	0.0001	52.3	45.01
GUCY1B3	M1	M	Tan et al., 2014	0.7886	28.8	50
GZMK	M1	M	Tan et al., 2014	3.20E-05	216.66	228.85
HAS2	M1	NA	dbEMT	0.4407	32.03	40.44
HMGA2	M1	NA	dbEMT	6.50E-08	37.2	33.64
HMOX1	M1	NA	dbEMT	0.0792	216.66	191.21
HNF4A	M1	NA	dbEMT	0.0247	38	62.36
HOXB7	M1	NA	dbEMT	0.0652	38.4	68.4
HOXB9	M1	NA	dbEMT	6.40E-10	51	46
HS3ST3B1	M1	NA	dbEMT	0.1448	68.75	37.64
HSPB2	M1	NA	dbEMT	0.0025	42	30.72
IGF1	M1	M	Tan et al., 2014	0.361	38	69.6
IL10RA	M1	M	Tan et al., 2014	0.2438	44.6	54.96
ITGA5	M1	NA	dbEMT	4.20E-07	27.96	48
ITGB1	M1	NA	dbEMT	5.30E-10	38	61
ITGB6	M1	E	Tan et al., 2014	0.4375	228.85	191.21
ITM2A	M1	M	Tan et al., 2014	6.00E-09	216.66	191.21
JAK2	M1	NA	dbEMT	0.0937	228.85	216.66
JAM2	M1	M	Tan et al., 2014	4.20E-12	228.85	216.66
JAM3	M1	M	Tan et al., 2014	0.0846	40.3	65.04
KCNH1	M1	NA	dbEMT	4.50E-08	55.66	43.56
KCNJ8	M1	M	Tan et al., 2014	1.20E-06	185.16	228.85
KDELC1	M1	M	Tan et al., 2014	0.0176	45	53.04
KIAA1462	M1	M	Tan et al., 2014	0.0024	228.85	191.21
KIT	M1	NA	dbEMT	6.60E-05	60.3	42
KRT8	M1	E	Tan et al., 2014	0.0053	37	66
LEF1	M1	NA	dbEMT	3.00E-07	105.6	171.43
LEP	M1	NA	dbEMT	0.0011	216.66	173.2
LEPRE1	M1	M	Tan et al., 2014	4.80E-09	228.85	191.21
LGALS1	M1	M	Tan et al., 2014	0.1714	47.28	49
LHFP	M1	M	Tan et al., 2014	0.5578	42.05	56
LIMS1	M1	NA	dbEMT	9.10E-05	31.2	42
MAGED1	M1	NA	dbEMT	9.00E-10	43	61
MAP1B	M1	M	Tan et al., 2014	3.90E-13	191.21	216.66
MDK	M1	NA	dbEMT	3.40E-08	49.02	48
MEOX2	M1	M	Tan et al., 2014	0.9971	228.85	191.21
MIR194-1	M1	NA	dbEMT	n/a	n/a	n/a
MIR34C	M1	NA	dbEMT	0.8392	57	43

MIR9-1	M1	NA	dbEMT	n/a	n/a	n/a
MKL1	M1	NA	dbEMT	0.05	56	40.8
MLPH	M1	E	Tan et al., 2014	0.0011	216.66	185.16
MMP14	M1	NA	dbEMT	1.90E-06	228.85	216.66
MMP3	M1	NA	dbEMT	6.60E-09	228.85	191.21
MMP9	M1	NA	dbEMT	0.9385	35	36.96
MSN	M1	M	Tan et al., 2014	0.8334	228.85	216.66
MUC4	M1	NA	dbEMT	0.0065	41.69	58.52
MXRA7	M1	M	Tan et al., 2014	0.0448	228.85	216.66
MYCN	M1	NA	dbEMT	0.3122	228.85	216.66
MYH14	M1	E	Tan et al., 2014	0.4864	228.85	216.66
MYL9	M1	M	Tan et al., 2014	1.30E-06	228.85	216.66
NAP1L3	M1	M	Tan et al., 2014	0.9801	228.85	216.66
NUAK1	M1	M	Tan et al., 2014	0.0218	216.66	184.04
OLFML2B	M1	M	Tan et al., 2014	0.0002	47	50
PAX2	M1	NA	dbEMT	0.6865	44	53.04
PCOLCE	M1	M	Tan et al., 2014	0.0134	216.66	228.85
PDZRN3	M1	M	Tan et al., 2014	0.0088	216.66	191.21
PIK3CA	M1	NA	dbEMT	0.0486	52.3	46.49
PLEKHO1	M1	M	Tan et al., 2014	0.0106	45	53.16
PLN	M1	M	Tan et al., 2014	2.00E-07	228.85	185.16
PMP22	M1	M	Tan et al., 2014	0.2577	216.66	228.85
POPDC3	M1	M	Tan et al., 2014	0.0073	32	39
POSTN	M1	NA	dbEMT	0.4699	46	50
PRKCA	M1	NA	dbEMT	0.0135	228.85	185.16
PTGIS	M1	M	Tan et al., 2014	0.3289	30	44.4
RAC1	M1	NA	dbEMT	0.0056	42	61
RARRES2	M1	M	Tan et al., 2014	0.0004	216.66	228.85
RDX	M1	NA	dbEMT	0.1448	185.16	216.66
RECK	M1	M	Tan et al., 2014	0.0155	228.85	216.66
RNF111	M1	NA	dbEMT	0.1137	44.61	52.3
ROCK1	M1	NA	dbEMT	0.4053	216.66	191.21
RUNX3	M1	NA	dbEMT	0.3587	39	63.6
SACS	M1	M	Tan et al., 2014	8.80E-06	228.85	216.66
SDC2	M1	M	Tan et al., 2014	1.10E-05	39	32.4
SERPINE1	M1	M	Tan et al., 2014	0.0335	228.85	216.66
SERPINF1	M1	M	Tan et al., 2014	0.0005	216.66	191.21
SFRP4	M1	M	Tan et al., 2014	0.003	228.85	216.66
SH2B3	M1	M	Tan et al., 2014	0.029	228.85	191.21
SHH	M1	NA	dbEMT	2.30E-05	216.66	185.16
SIX1	M1	NA	dbEMT	1.30E-08	171.43	216.66
SMAD7	M1	NA	dbEMT	4.20E-08	228.85	216.66
SNAI1	M1	M	Tan et al., 2014	0.0042	216.66	191.21
SPARC	M1	M	Tan et al., 2014	0.1208	184.04	216.66
SRC	M1	NA	dbEMT	0.0673	37.35	72.49
SRPX	M1	M	Tan et al., 2014	0.0101	49	47.51
STAT5A	M1	NA	dbEMT	3.30E-08	44	57
STAT5B	M1	NA	dbEMT	0.9245	38.57	60
STON1	M1	M	Tan et al., 2014	1.00E-16	228.85	216.66
SYNE1	M1	M	Tan et al., 2014	1.00E-16	38	32

SYT11	M1	M	Tan et al., 2014	0.0444	40.08	61.05
TAGLN	M1	M	Tan et al., 2014	1.80E-12	171.43	216.66
TCF3	M1	NA	dbEMT	1.20E-13	216.66	191.21
TFF3	M1	E	Tan et al., 2014	0.8548	39	32.82
TGFB1I1	M1	M	Tan et al., 2014	0.0044	228.85	216.66
TGFBR1	M1	NA	dbEMT	0.0005	216.66	191.21
TGM2	M1	NA	dbEMT	0.973	216.66	228.85
TM4SF5	M1	NA	dbEMT	0.7866	228.85	185.16
TMEFF1	M1	M	Tan et al., 2014	5.70E-06	191.21	228.85
TMPRSS2	M1	E	Tan et al., 2014	2.80E-09	216.66	191.21
TNC	M1	M	Tan et al., 2014	0.4698	228.85	191.21
TNS1	M1	M	Tan et al., 2014	0.1884	50.3	47.4
TOX3	M1	E	Tan et al., 2014	0.0972	216.66	191.21
TPM2	M1	M	Tan et al., 2014	0.0012	228.85	216.66
TRPC1	M1	M	Tan et al., 2014	0.176	216.66	228.85
TSPAN8	M1	E	Tan et al., 2014	1.10E-07	228.85	185.16
TUBA1A	M1	M	Tan et al., 2014	0.0045	228.85	216.66
VCAM1	M1	M	Tan et al., 2014	0.0004	228.85	191.21
VSIG4	M1	M	Tan et al., 2014	2.40E-07	26	45
WIPF1	M1	M	Tan et al., 2014	0.7863	24.07	56.4
WNT1	M1	NA	dbEMT	0.1527	184.04	216.66
WNT3A	M1	NA	dbEMT	n/a	n/a	n/a
WWTR1	M1	M	Tan et al., 2014	0.8466	228.85	216.66
ACVR1	M2	NA	dbEMT	9.70E-05	228.85	216.66
BICC1	M2	M	Tan et al., 2014	0.0017	228.85	216.66
BNC2	M2	M	Tan et al., 2014	0.2897	49.2	47
BRAF	M2	NA	dbEMT	1.00E-10	60	39.95
C1R	M2	M	Tan et al., 2014	9.20E-09	228.85	216.66
C1S	M2	M	Tan et al., 2014	1.30E-13	228.85	216.66
CDH13	M2	NA	dbEMT	1.20E-06	228.85	191.21
CDH2	M2	M	Tan et al., 2014	0.6864	43	55.43
CDKN1B	M2	NA	dbEMT	0.011	228.85	216.66
CDKN2A	M2	NA	dbEMT	0.0142	43	56
CEP170	M2	M	Tan et al., 2014	0.3789	216.66	228.85
COL5A2	M2	M	Tan et al., 2014	0.0009	29	41.95
CTGF	M2	NA	dbEMT	0.218	191.21	216.66
DAB2	M2	NA	dbEMT	0.7456	228.85	216.66
DCN	M2	M	Tan et al., 2014	0.0188	216.66	191.21
DNAJB6	M2	NA	dbEMT	0.005	29	46
EMP3	M2	M	Tan et al., 2014	0.022	44	54.7
ENG	M2	NA	dbEMT	0.0007	38	68.48
ERF	M2	NA	dbEMT	9.10E-05	228.85	216.66
FBN1	M2	M	Tan et al., 2014	2.60E-05	216.66	191.21
FN1	M2	M	Tan et al., 2014	9.00E-05	228.85	185.16
FSTL1	M2	M	Tan et al., 2014	0.0951	50	47.28
FYN	M2	M	Tan et al., 2014	0.0694	57	40
GIPC2	M2	NA	dbEMT	0.0005	228.85	216.66
GJA1	M2	M	Tan et al., 2014	2.30E-05	228.85	216.66
HDAC6	M2	NA	dbEMT	0.0142	46	50.86
HEG1	M2	M	Tan et al., 2014	5.40E-11	48	48

HIF1A	M2	NA	dbEMT	0.0338	40.44	60
HSPB1	M2	NA	dbEMT	6.30E-13	51	47
IGF1R	M2	NA	dbEMT	2.20E-09	191.21	216.66
IGFBP5	M2	M	Tan et al., 2014	0.8651	52.32	46
KDM3A	M2	NA	dbEMT	0.0746	43	57
KLF4	M2	NA	dbEMT	2.20E-05	228.85	216.66
LOX	M2	M	Tan et al., 2014	0.0225	216.66	191.21
MAF	M2	M	Tan et al., 2014	0.7329	191.21	216.66
MAP3K4	M2	NA	dbEMT	0.6733	34	37.52
MAP3K7	M2	NA	dbEMT	3.50E-07	50	47
MCAM	M2	NA	dbEMT	0.4734	185.16	216.66
MIR21	M2	NA	dbEMT	0.4656	33.36	76
MMP13	M2	NA	dbEMT	0.9199	40.56	58
MMP2	M2	M	Tan et al., 2014	0.0012	44.61	53.1
MOXD1	M2	M	Tan et al., 2014	6.20E-07	228.85	216.66
MPDZ	M2	M	Tan et al., 2014	0.0239	31.51	38
NCSTN	M2	NA	dbEMT	0.0007	216.66	191.21
NOG	M2	NA	dbEMT	0.2196	45	55.9
NR3C1	M2	M	Tan et al., 2014	0.4995	42	60
OLFML3	M2	M	Tan et al., 2014	3.40E-08	216.66	228.85
PDE4A	M2	NA	dbEMT	1.00E-16	216.66	228.85
PDGFC	M2	M	Tan et al., 2014	0.0002	228.85	216.66
PLXNB2	M2	E	Tan et al., 2014	0.1499	28	46.8
POU5F1	M2	NA	dbEMT	0.0007	216.66	228.85
PTEN	M2	NA	dbEMT	0.6247	173.2	216.66
PTGDS	M2	M	Tan et al., 2014	1.40E-07	50	46.68
PTGS2	M2	NA	dbEMT	0.0019	26	48
PTN	M2	NA	dbEMT	0.0008	51	46
PTX3	M2	M	Tan et al., 2014	0.3181	31.51	42
RGS2	M2	M	Tan et al., 2014	0.6106	228.85	216.66
ROR2	M2	NA	dbEMT	0.0039	48	48
RUNX1T1	M2	M	Tan et al., 2014	1.30E-07	37.64	34
SAMSN1	M2	M	Tan et al., 2014	0.8113	43.56	58
SEMA3E	M2	NA	dbEMT	6.60E-08	25	49.2
SEMA4C	M2	NA	dbEMT	0.932	228.85	185.16
SMAD4	M2	NA	dbEMT	4.10E-08	184.04	216.66
SNAI2	M2	M	Tan et al., 2014	0.2975	228.85	216.66
SOAT1	M2	M	Tan et al., 2014	0.0003	228.85	216.66
SPOCK1	M2	M	Tan et al., 2014	0.9936	228.85	216.66
SRF	M2	NA	dbEMT	0.6437	28.85	216.66
STAT3	M2	NA	dbEMT	0.1408	115	171.43
SYNM	M2	M	Tan et al., 2014	0.001	35	37.64
TAB1	M2	NA	dbEMT	n/a	n/a	n/a
TBX3	M2	NA	dbEMT	1.80E-10	228.85	216.66
TCF4	M2	M	Tan et al., 2014	0.9205	29	43
TP53	M2	NA	dbEMT	0.0005	216.66	191.21
TRPS1	M2	NA	dbEMT	0.2083	28.96	44
VCAN	M2	M	Tan et al., 2014	0.2395	228.85	216.66
VIM	M2	M	Tan et al., 2014	0.0949	185.16	216.66
WNT5A	M2	NA	dbEMT	0.433	228.85	216.66

ZEB1	M2	M	Tan et al., 2014	1.10E-06	216.66	228.85
ZEB2	M2	M	Tan et al., 2014	0.0284	44	54
ZFPM2	M2	M	Tan et al., 2014	0.0002	216.66	228.85
AKAP12	M3	M	Tan et al., 2014	0.0004	34.8	37
ANPEP	M3	NA	dbEMT	0.0793	228.85	216.66
AR	M3	NA	dbEMT	4.20E-05	44.6	53.04
ASPN	M3	M	Tan et al., 2014	0.2608	228.85	191.21
AXL	M3	M	Tan et al., 2014	0.0004	27	45.6
BGN	M3	M	Tan et al., 2014	0.721	43.7	56.04
BMI1	M3	NA	dbEMT	0.7753	228.85	216.66
BMP4	M3	NA	dbEMT	0.058	43	56.58
CCL8	M3	M	Tan et al., 2014	0.0025	23.82	61
CMTM8	M3	NA	dbEMT	0.9132	216.66	191.21
COL8A1	M3	NA	dbEMT	5.40E-06	42.96	55.43
CTSZ	M3	NA	dbEMT	0.0819	228.85	216.66
CYP1B1	M3	M	Tan et al., 2014	0.563	54.77	44
DDX5	M3	NA	dbEMT	3.30E-05	28.8	43.7
DLX4	M3	NA	dbEMT	0.0059	228.85	216.66
EDNRA	M3	NA	dbEMT	0.7264	228.85	191.21
EFEMP1	M3	M	Tan et al., 2014	0.013	48	48
EPB41L5	M3	NA	dbEMT	1.00E-16	147	171.43
ERBB2IP	M3	NA	dbEMT	0.0018	50	47
EVI2A	M3	M	Tan et al., 2014	0.0026	26	49.2
FBLN1	M3	M	Tan et al., 2014	3.60E-05	216.66	228.85
FERMT2	M3	M	Tan et al., 2014	0.0285	216.66	228.85
FGFR1	M3	NA	dbEMT	0.0002	228.85	216.66
FOXC2	M3	E	Tan et al., 2014	0.0427	30	40.71
FXYD6	M3	M	Tan et al., 2014	0.0003	228.85	216.66
GAS1	M3	M	Tan et al., 2014	0.3391	228.85	216.66
GLIPR1	M3	M	Tan et al., 2014	1.80E-05	184.04	216.66
GLRX	M3	NA	dbEMT	0.9264	228.85	216.66
GLYR1	M3	M	Tan et al., 2014	1.30E-09	31.18	40.44
GNG11	M3	M	Tan et al., 2014	0.0043	228.85	191.21
GPM6B	M3	M	Tan et al., 2014	0.2889	48	48
HGF	M3	NA	dbEMT	0.0058	30	40.8
HNMT	M3	E	Tan et al., 2014	0.0082	53.1	44.61
HOXA10	M3	NA	dbEMT	0.038	42	59
IGFBP3	M3	NA	dbEMT	0.0446	38	65.1
ISLR	M3	M	Tan et al., 2014	0.0464	52.54	44.22
LAMA5	M3	NA	dbEMT	0.0002	191.21	228.85
LRP6	M3	NA	dbEMT	0.006	36	36.04
MAFB	M3	M	Tan et al., 2014	0.1534	29	42
MANSC1	M3	E	Tan et al., 2014	0.6974	228.85	216.66
MAP3K3	M3	NA	dbEMT	0.0029	44	54
MAPK14	M3	NA	dbEMT	0.0918	40.3	57.3
MAPK7	M3	NA	dbEMT	0.0005	37	34.8
MFAP4	M3	M	Tan et al., 2014	5.80E-09	43	58
MIR15B	M3	NA	dbEMT	n/a	n/a	n/a
MIR193A	M3	NA	dbEMT	n/a	n/a	n/a
MIR34A	M3	NA	dbEMT	n/a	n/a	n/a

MMP7	M3	NA	dbEMT	0.096	129	171.43
MUC16	M3	NA	dbEMT	0.9594	47	50
MYLK	M3	M	Tan et al., 2014	0.7165	54	43
NFIC	M3	NA	dbEMT	0.0253	216.66	228.85
PLXNC1	M3	M	Tan et al., 2014	0.2577	184.04	216.66
PLXND1	M3	NA	dbEMT	0.0492	35	36.9
PPARG	M3	NA	dbEMT	0.0007	51	45.84
PTPN14	M3	NA	dbEMT	0.4434	35.98	71
PTRF	M3	M	Tan et al., 2014	0.0004	216.66	228.85
QKI	M3	M	Tan et al., 2014	2.00E-05	29	42
RAF1	M3	NA	dbEMT	0.2984	228.85	216.66
ROCK2	M3	NA	dbEMT	0.0786	228.85	216.66
S100A4	M3	NA	dbEMT	0.0437	216.66	228.85
SERPING1	M3	M	Tan et al., 2014	1.30E-08	35	36.9
SOBP	M3	M	Tan et al., 2014	0.6293	51	45.84
SON	M3	NA	dbEMT	0.6585	35.98	71
SPARCL1	M3	M	Tan et al., 2014	1.00E-13	216.66	228.85
SRGN	M3	M	Tan et al., 2014	0.2041	29	42
TEAD1	M3	NA	dbEMT	0.0005	228.85	216.66
TGFB2	M3	NA	dbEMT	0.3875	228.85	216.66
TGFB3	M3	NA	dbEMT	1.50E-11	228.85	216.66
TIMP1	M3	NA	dbEMT	0.0414	228.85	216.66
TMEM158	M3	M	Tan et al., 2014	0.0014	57.27	41.64
TRIM31	M3	E	Tan et al., 2014	3.40E-06	191.21	216.66
TWIST1	M3	M	Tan et al., 2014	0.7013	52	46
TWIST2	M3	M	Tan et al., 2014	0.0003	30	43
VWCE	M3	NA	dbEMT	0.1218	34.82	37
WISP2	M3	NA	dbEMT	0.0002	40.8	58
WNT6	M3	NA	dbEMT	0.0979	228.85	216.66
ZCCHC24	M3	M	Tan et al., 2014	7.40E-12	36	71.04
BAG2	Other M	M	Tan et al., 2014	0.6068	52.2	45.9
CSNK2B	Other M	NA	dbEMT	1.20E-09	65.04	37.35
CTBP1	Other M	NA	dbEMT	0.5356	191.21	216.66
HMGB3	Other M	NA	dbEMT	2.60E-08	48	27.4
KHDRBS1	Other M	NA	dbEMT	0.0008	58.52	41.65
LEFTY1	Other M	NA	dbEMT	1.60E-08	228.85	216.66
MAPK1	Other M	NA	dbEMT	0.1309	35.98	36.9
MS4A4A	Other M	M	Tan et al., 2014	0.496	36	35.98
MS4A6A	Other M	M	Tan et al., 2014	0.0004	216.66	228.85
MTDH	Other M	NA	dbEMT	3.50E-10	216.66	184.04
NF1	Other M	NA	dbEMT	3.00E-11	191.21	216.66

Supplementary Table 3 Enrichment of EMT genes among gene upregulated by EMT regulators in Tauble et al.

**Down Regulated Genes (FC < 2)**					
	TGF-β	Twist	Snail	Gsc	sh-Ecad
E	78 (*p* = 2.1e-61)	110 (*p* = 4.8e-80)	116 (*p* = 1.1e-77)	114 (*p* = 1.9e-55)	148 (*p* = 1.9e-94)
M	3 (*p* = 0.47)	7 (*p* = 0.67)	6 (*p* = 0.47)	8 (*p* = 0.41)	14 (*p* = 0.78)
**Up Regulated Genes (FC > 2)**					
	TGF-β	Twist	Snail	Gsc	sh-Ecad
E	7 (*p* = 1)	12 (*p* = 0.77)	12 (*p* = 1)	21 (*p* = 0.82)	4 (*p* = 1)
M	70 (*p* = 1.1e-51)	88 (*p* = 7.7e-52)	94 (*p* = 1.5e-61)	103 (*p* = 1.3e-45)	82 (*p* = 2.8e-48)
M1	35 (*p* = 2.8e-25)	43 (*p* = 1.9e-24)	46 (*p* = 6.1e-29)	40 (*p* = 1.3e-13)	41 (*p* = 9.7e-24)
M2	23 (*p* = 6.1e-21)	24 (*p* = 1.9e-16)	28 (*p* = 5.5e-22)	37 (*p* = 1.1e-24)	23 (*p* = 4.5e-16)
M3	12 (*p* = 2.4e-8)	21 (*p* = 6.4e-14)	20 (*p* = 1.7e-13)	26 (*p* = 4.9e-14)	18 (*p* = 2.1e-11)

All *p* values are based on Fisher’s exact test.

Supplementary Table 4. List of GO Terms enriched in all epithelial genes

GO	P.adjust	Short Description
GO:0016324	2.05E-08	apical plasma membrane
GO:0016328	3.61E-08	lateral plasma membrane
GO:0005911	7.14E-08	cell-cell junction
GO:0016323	9.88E-08	basolateral plasma membrane
GO:0070268	1.95E-07	cornification
GO:0007219	1.95E-07	Notch signaling pathway
GO:0030057	4.83E-07	desmosome
GO:0098609	2.15E-06	cell-cell adhesion
GO:0030216	3.08E-06	keratinocyte differentiation
GO:0005923	4.08E-06	bicellular tight junction
GO:0016477	4.08E-06	cell migration
GO:0071944	9.66E-06	cell periphery
GO:0007179	1.77E-05	transforming growth factor beta receptor signaling pathway
GO:0008013	2.55E-05	beta-catenin binding
GO:0001934	4.79E-05	positive regulation of protein phosphorylation
GO:0001533	5.06E-05	cornified envelope
GO:0050679	5.06E-05	positive regulation of epithelial cell proliferation
GO:0043406	5.66E-05	positive regulation of MAP kinase activity
GO:0090303	5.66E-05	positive regulation of wound healing
GO:0002934	6.03E-05	desmosome organization
GO:0051897	8.28E-05	positive regulation of protein kinase B signaling
GO:0060749	8.28E-05	mammary gland alveolus development
GO:0005913	8.41E-05	cell-cell adherens junction
GO:0007173	0.000113066	epidermal growth factor receptor signaling pathway
GO:0016327	0.000113066	apicolateral plasma membrane
GO:0050839	0.000152801	cell adhesion molecule binding
GO:0005829	0.000156763	cytosol
GO:0038132	0.000209644	neuregulin binding
GO:0086083	0.000209644	cell adhesive protein binding involved in bundle of His cell-Purkinje myocyte communication
GO:0038128	0.000283371	ERBB2 signaling pathway
GO:0061436	0.000304381	establishment of skin barrier
GO:0098911	0.000308222	regulation of ventricular cardiac muscle cell action potential
GO:0000165	0.000315781	MAPK cascade
GO:0043235	0.000409511	receptor complex
GO:0008544	0.000455941	epidermis development
GO:0071773	0.000497706	cellular response to BMP stimulus
GO:0070830	0.000497706	bicellular tight junction assembly
GO:0005154	0.000497706	epidermal growth factor receptor binding

GO:0086073	0.000501582	bundle of His cell-Purkinje myocyte adhesion involved in cell communication
GO:0014704	0.000552585	intercalated disc
GO:0009925	0.000583926	basal plasma membrane
GO:0031528	0.000643276	microvillus membrane
GO:0005887	0.001074063	integral component of plasma membrane
GO:1900740	0.001076022	positive regulation of protein insertion into mitochondrial membrane involved in apoptotic signaling pathway
GO:0005198	0.001308149	structural molecule activity
GO:0060672	0.001534535	epithelial cell morphogenesis involved in placental branching
GO:0032496	0.001561501	response to lipopolysaccharide
GO:0048709	0.001580009	oligodendrocyte differentiation
GO:0032091	0.001580009	negative regulation of protein binding
GO:0032147	0.001880503	activation of protein kinase activity
GO:0070372	0.001880503	regulation of ERK1 and ERK2 cascade
GO:0070371	0.001880503	ERK1 and ERK2 cascade
GO:0045121	0.001962464	membrane raft
GO:0008134	0.002462225	transcription factor binding
GO:0071437	0.002820133	invadopodium
GO:0030054	0.002820133	cell junction
GO:0045747	0.003147601	positive regulation of Notch signaling pathway
GO:0031424	0.003147601	keratinization
GO:0042127	0.003362169	regulation of cell population proliferation
GO:0030674	0.003536732	protein binding, bridging
GO:0005200	0.00376746	structural constituent of cytoskeleton
GO:0098641	0.003807955	cadherin binding involved in cell-cell adhesion
GO:0023019	0.003807955	signal transduction involved in regulation of gene expression
GO:0045216	0.003807955	cell-cell junction organization
GO:0043547	0.004558012	positive regulation of GTPase activity
GO:0005112	0.00490046	Notch binding
GO:0030855	0.005509904	epithelial cell differentiation
GO:0046934	0.006037726	phosphatidylinositol-4,5-bisphosphate 3-kinase activity
GO:0051496	0.006466229	positive regulation of stress fiber assembly
GO:0048754	0.007617998	branching morphogenesis of an epithelial tube
GO:0001228	0.007830327	DNA-binding transcription activator activity, RNA polymerase II-specific
GO:0034332	0.008195817	adherens junction organization
GO:0086091	0.008195817	regulation of heart rate by cardiac conduction
GO:2000146	0.008590302	negative regulation of cell motility
GO:0019899	0.008836847	enzyme binding
GO:0090263	0.008841438	positive regulation of canonical Wnt signaling pathway
GO:0010468	0.008841438	regulation of gene expression
GO:0042104	0.008841438	positive regulation of activated T cell proliferation

GO:0031982	0.009001695	vesicle
GO:0071504	0.009449414	cellular response to heparin
GO:0030618	0.009449414	transforming growth factor beta receptor, pathway-specific cytoplasmic mediator activity
GO:0009967	0.010621008	positive regulation of signal transduction
GO:0034236	0.011122653	protein kinase A catalytic subunit binding
GO:0030154	0.012686211	cell differentiation
GO:0051384	0.012972101	response to glucocorticoid
GO:0010469	0.013970766	regulation of signaling receptor activity
GO:0030307	0.014460919	positive regulation of cell growth
GO:0051017	0.014460919	actin filament bundle assembly
GO:0045668	0.014460919	negative regulation of osteoblast differentiation
GO:0044319	0.014460919	wound healing, spreading of cells
GO:0060487	0.016323064	lung epithelial cell differentiation
GO:1902949	0.016323064	positive regulation of tau-protein kinase activity
GO:0060056	0.016323064	mammary gland involution
GO:0045840	0.016644153	positive regulation of mitotic nuclear division
GO:2000145	0.016644153	regulation of cell motility
GO:0005882	0.017306919	intermediate filament
GO:0030949	0.017306919	positive regulation of vascular endothelial growth factor receptor signaling pathway
GO:0001658	0.017306919	branching involved in ureteric bud morphogenesis
GO:0022408	0.017306919	negative regulation of cell-cell adhesion
GO:0060441	0.017306919	epithelial tube branching involved in lung morphogenesis
GO:2000060	0.017306919	positive regulation of ubiquitin-dependent protein catabolic process
GO:0035326	0.017306919	enhancer binding
GO:0048471	0.017395121	perinuclear region of cytoplasm
GO:0072659	0.018286409	protein localization to plasma membrane
GO:0004888	0.018646687	transmembrane signaling receptor activity
GO:0051781	0.021713298	positive regulation of cell division
GO:0044212	0.021713298	transcription regulatory region DNA binding
GO:0007229	0.023968789	integrin-mediated signaling pathway
GO:0071141	0.023968789	SMAD protein complex
GO:0035655	0.023968789	interleukin-18-mediated signaling pathway
GO:0050731	0.023968789	positive regulation of peptidyl-tyrosine phosphorylation
GO:0060687	0.023968789	regulation of branching involved in prostate gland morphogenesis
GO:0005138	0.023968789	interleukin-6 receptor binding
GO:0030056	0.023968789	hemidesmosome
GO:0045617	0.023968789	negative regulation of keratinocyte differentiation
GO:0061384	0.023968789	heart trabecula morphogenesis
GO:0043231	0.023981011	intracellular membrane-bounded organelle
GO:0007166	0.024190272	cell surface receptor signaling pathway

GO:0042531	0.024911995	positive regulation of tyrosine phosphorylation of STAT protein
GO:0030676	0.025334535	Rac guanyl-nucleotide exchange factor activity
GO:2000249	0.025334535	regulation of actin cytoskeleton reorganization
GO:0008584	0.025892253	male gonad development
GO:0007565	0.025892253	female pregnancy
GO:0046777	0.027322274	protein autophosphorylation
GO:0048557	0.030290213	embryonic digestive tract morphogenesis
GO:0006970	0.030290213	response to osmotic stress
GO:0031663	0.030290213	lipopolysaccharide-mediated signaling pathway
GO:0005044	0.030290213	scavenger receptor activity
GO:0030279	0.030290213	negative regulation of ossification
GO:0000187	0.032035464	activation of MAPK activity
GO:0071144	0.032035464	heteromeric SMAD protein complex
GO:0061302	0.032035464	smooth muscle cell-matrix adhesion
GO:0038092	0.032035464	nodal signaling pathway
GO:0043588	0.032035464	skin development
GO:0004450	0.032035464	isocitrate dehydrogenase (NADP+) activity
GO:0006097	0.032035464	glyoxylate cycle
GO:0048340	0.032035464	paraxial mesoderm morphogenesis
GO:0070878	0.032035464	primary miRNA binding
GO:0010482	0.032035464	regulation of epidermal cell division
GO:0062043	0.032035464	positive regulation of cardiac epithelial to mesenchymal transition
GO:0001773	0.032035464	myeloid dendritic cell activation
GO:0071639	0.032035464	positive regulation of monocyte chemotactic protein-1 production
GO:0003169	0.032035464	coronary vein morphogenesis
GO:0022612	0.032035464	gland morphogenesis
GO:0005172	0.032035464	vascular endothelial growth factor receptor binding
GO:0071506	0.032035464	cellular response to mycophenolic acid
GO:0005088	0.032963162	Ras guanyl-nucleotide exchange factor activity
GO:0007266	0.033093313	Rho protein signal transduction
GO:0043034	0.033813585	costamere
GO:0008283	0.035449084	cell proliferation
GO:0032570	0.035449084	response to progesterone
GO:0046332	0.036079675	SMAD binding
GO:0032755	0.039660936	positive regulation of interleukin-6 production
GO:0005070	0.039660936	SH3/SH2 adaptor activity
GO:0045104	0.039843233	intermediate filament cytoskeleton organization
GO:0033627	0.039843233	cell adhesion mediated by integrin
GO:0001889	0.040235914	liver development
GO:0005794	0.040713061	Golgi apparatus
GO:0045741	0.041122468	positive regulation of epidermal growth factor-activated receptor activity
GO:0035313	0.041122468	wound healing, spreading of epidermal cells

GO:0060253	0.041122468	negative regulation of glial cell proliferation
GO:0032872	0.041122468	regulation of stress-activated MAPK cascade
GO:0032835	0.041122468	glomerulus development
GO:0033689	0.041122468	negative regulation of osteoblast proliferation
GO:0004714	0.041122468	transmembrane receptor protein tyrosine kinase activity
GO:0005912	0.041122468	adherens junction
GO:0048469	0.042765817	cell maturation
GO:0001843	0.044774075	neural tube closure
GO:0043407	0.04804062	negative regulation of MAP kinase activity

Supplementary Table 5. List of GO Terms enriched in epithelial genes which are differentially expressed in response to both TGF-β and ZEB1.

GO	P.adjust	Short Description
GO:0070062	2.82E-08	extracellular exosome
GO:0005886	0.00021	plasma membrane
GO:0070268	0.00038	cornification
GO:0060672	0.000546	epithelial cell morphogenesis involved in placental branching
GO:0045296	0.000617	cadherin binding
GO:0050839	0.000617	cell adhesion molecule binding
GO:0098609	0.000617	cell-cell adhesion
GO:0002934	0.000968	desmosome organization
GO:0016323	0.000968	basolateral plasma membrane
GO:0005923	0.003392	bicellular tight junction
GO:0098641	0.003531	cadherin binding involved in cell-cell adhesion
GO:0030057	0.010324	desmosome
GO:0005913	0.010391	cell-cell adherens junction
GO:0016328	0.010391	lateral plasma membrane
GO:0005911	0.011385	cell-cell junction
GO:0005154	0.021427	epidermal growth factor receptor binding
GO:0031424	0.031024	keratinization
GO:0034332	0.031024	adherens junction organization
GO:0001843	0.03743	neural tube closure
GO:0072659	0.040151	protein localization to plasma membrane

Supplementary Table 6. Gene sets enriched in M-gene clusters

M-gene Cluster	GSEA Group	GSEA Group Description	q-value
M1	Invasive Ductal Cancer (up)	Genes up-regulated in invasive ductal carcinoma (IDC) relative to ductal carcinoma in situ (DCIS, non-invasive).	3.96E-55
M1	Cluster 11b urothelial carcinoma (up)	Genes specifically up-regulated in Cluster IIb of urothelial cell carcinom (UCC) tumors.	3.04E-43
M1	Luminal vs mesenchymal breast cancer (down)	Genes down-regulated in luminal-like breast cancer cell lines compared to the mesenchymal-like ones.	1.44E-30
M1	Luminal B breast cancer (down)	Genes down-regulated in the luminal B subtype of breast cancer.	5.57E-29
M1	Prostate cancer (down)	Genes down-regulated in prostate cancer samples.	1.17E-28
M1	Kras2La mouse lung cancer (down)	Genes down-regulated in the Kras2LA mouse lung cancer model with mutated KRAS [GeneID=3845].	5.77E-26
M1	Norma-like breast cancer (up)	Genes up-regulated in the normal-like subtype of breast cancer.	4.41E-21
M1	Papillary thyroid carcinoma (down)	Genes down-regulated in papillary thyroid carcinoma (PTC) compared to normal tissue.	1.68E-17
M1	S1 hepatocellular carcinoma: WNT signalling	Genes from 'subtype S1' signature of hepatocellular carcinoma (HCC): aberrant activation of the WNT signaling pathway.	2.38E-17
M1	Invasive signature from microenvironemental interaction	Invasiveness signature resulting from cancer cell/microenvironment interaction.	4.98E-17
M1	Advanced vs. early gastric cancer (up)	Up-regulated genes distinguishing between two subtypes of gastric cancer: advanced (AGC) and early (EGC).	3.73E-14
M1	Ovarian tumor suboptimal debulk (up)	Genes whose expression in suboptimally debulked ovarian tumors is associated with survival prognosis.	1.12E-11
M1	Pancreatic ductal adenocarcinoma (up)	Genes up-regulated in pancreatic ductal adenocarcinoma (PDAC) identified in a meta analysis across four independent studies.	5.72E-11
M1	Basal breast cancer (up)	Genes up-regulated in basal subtype of breast cancer samles.	5.23E-10
M1	Luminal vs basal breast cancer (down)	Genes down-regulated in luminal-like breast cancer cell lines compared to the basal-like ones.	1.47E-09
M1	Basal breast cancer (down)	Genes down-regulated in basal subtype of breast cancer samles.	1.68E-09
M1	Stromal co-expression in breast cancer	Cluster 4: selected stromal genes clustered together across breast cancer samples.	3.04E-09

M1	ADHC breast cancer (up)	Genes up-regulated in atypical ductal hyperplastic tissues from patients with (ADHC) breast cancer vs those without the cancer (ADH).	4.53E-09
M2	Invasive Ductal Cancer (up)	Genes up-regulated in invasive ductal carcinoma (IDC) relative to ductal carcinoma in situ (DCIS, non-invasive).	9.18E-34
M2	Luminal vs mesenchymal breast cancer (down)	Genes down-regulated in luminal-like breast cancer cell lines compared to the mesenchymal-like ones.	4.09E-24
M2	Cluster 11b urothelial carcinoma (up)	Genes specifically up-regulated in Cluster IIb of urothelial cell carcinom (UCC) tumors.	1.05E-13
M2	Advanced vs. early gastric cancer (up)	Up-regulated genes distinguishing between two subtypes of gastric cancer: advanced (AGC) and early (EGC).	1.05E-13
M2	Luminal vs basal breast cancer (down)	Genes down-regulated in luminal-like breast cancer cell lines compared to the basal-like ones.	6.19E-13
M2	Invasive signature from microenvironemental interaction	Invasiveness signature resulting from cancer cell/microenvironment interaction.	6.19E-13
M2	Pancreatic ductal adenocarcinoma (up)	Genes up-regulated in pancreatic ductal adenocarcinoma (PDAC) identified in a meta analysis across four independent studies.	2.43E-10
M2	Mucinous ovarian carcinoma (down)	Genes down-regulated in mucinous ovarian carcinoma tumors of low malignant potential (LMP) compared to normal ovarian surface epithelium tissue.	3.49E-10
M2	Prostate cancer (down)	Genes down-regulated in prostate cancer samples.	5.29E-09
M2	S1 hepatocellular carcinoma: WNT signaling	Genes from 'subtype S1' signature of hepatocellular carcinoma (HCC): aberrant activation of the WNT signaling pathway.	3.13E-08
M2	Luminal B breast cancer (down)	Genes down-regulated in the luminal B subtype of breast cancer.	2.58E-07
M2	Norma-like breast cancer (up)	Genes up-regulated in the normal-like subtype of breast cancer.	6.14E-07
M2	Ovarian tumor suboptimal debulk (up)	Genes whose expression in suboptimally debulked ovarian tumors is associated with survival prognosis.	1.05E-06
M2	Serrated vs conventional colorectal cancer (up)	Genes up-regulated in serrated vs conventional colorectal carcinoma (CRC) samples.	1.96E-06
M2	Tumor methylation	Genes whose DNA was methylated both in primary tumors and across a panel of cancer cell lines.	2.05E-06
M2	Kras2La mouse lung cancer (down)	Genes down-regulated in the Kras2LA mouse lung cancer model with mutated KRAS [GeneID=3845].	2.81E-06

M3	Invasive Ductal Cancer (up)	Genes up-regulated in invasive ductal carcinoma (IDC) relative to ductal carcinoma in situ (DCIS, non-invasive).	1.68E-25
M3	Cluster 11b urothelial carcinoma (up)	Genes specifically up-regulated in Cluster IIb of urothelial cell carcinom (UCC) tumors.	2.69E-15
M3	Advanced vs. early gastric cancer (up)	Up-regulated genes distinguishing between two subtypes of gastric cancer: advanced (AGC) and early (EGC).	4.03E-12
M3	Luminal B breast cancer (down)	Genes down-regulated in the luminal B subtype of breast cancer.	7.93E-12
M3	Luminal vs mesenchymal breast cancer (down)	Genes down-regulated in luminal-like breast cancer cell lines compared to the mesenchymal-like ones.	9.19E-12
M3	Medullary vs ductal breast cancer (down)	Genes down-regulated in medullary breast cancer (MBC) relative to ductal breast cancer (DBD).	2.67E-09
M3	Kras2La mouse lung cancer (down)	Genes down-regulated in the Kras2LA mouse lung cancer model with mutated KRAS [GeneID=3845].	2.77E-08
M3	Prostate cancer (down)	Genes down-regulated in prostate cancer samples.	6.46E-08
M3	Basal breast cancer (down)	Genes down-regulated in basal subtype of breast cancer samles.	1.96E-07
M3	African-American vs. European-American (up)	Genes up-regulated in prostate cancer samples from African-American patients compared to those from the European-American patients.	1.96E-07
M3	Norma-like breast cancer (up)	Genes up-regulated in the normal-like subtype of breast cancer.	6.73E-07
M3	Mucinous ovarian carcinoma (down)	Genes down-regulated in mucinous ovarian carcinoma tumors of low malignant potential (LMP) compared to normal ovarian surface epithelium tissue.	2.90E-06
M3	Basal breast cancer (up)	Genes up-regulated in basal subtype of breast cancer samles.	6.84E-06
M3	Hhigh grade prostatic intraepithelial neoplasia (up)	Early prostate development genes (up-regulated at 48 hr dihydrotestosterone [PubChem=10635]) which are also up-regulated in high grade prostatic intraepithelial neoplasia (PIN) vs invasive cancer.	1.40E-05
M3	Circulating endothelial cells from cancer patients (up)	Genes up-regulated in circulating endothelial cells (CEC) from cancer patients compared to those from healthy donors.	1.47E-05
M3	Luminal A breast cancer (up)	Genes up-regulated in the luminal A subtype of breast cancer.	1.56E-05
M3	CTNNB1 hepatocellular carcinoma (down)	Top 200 marker genes down-regulated in the 'CTNNB1' subclass of hepatocellular carcinoma (HCC); characterized by activated CTNNB1 [GeneID=1499].	1.97E-05

M3	Prostate cancer vs. benign prostate tumor (down)	Genes down-regulated in prostate cancer vs benign prostate tissue, based on a meta-analysis of five gene expression profiling studies.	2.76E-05
M3	Papillary thyroid carcinoma (up)	Genes up-regulated in papillary thyroid carcinoma (PTC) compared to normal tissue.	3.16E-05
M3	Brain stem cell cancer signature	Genes from the brain cancer stem (cancer stem cell, CSC) signature.	3.90E-05
M3	Onocytic follicular vs mitochondrial rich papillary thyroid cancer (down)	Genes down-regulated in oncocytic follicular carcinoma (FTC) vs mitochondrial-rich papillary carcinoma (PTC) types of thyroid cancer.	1.07E-04
M3	Invasive signature from microenvironemental interaction	Invasiveness signature resulting from cancer cell/microenvironment interaction.	1.39E-04

Supplementary Table 7. Genes from M-gene clusters overlapping with enriched cancer gene sets

M-gene Cluster	GSEA Group	Genes
M1	Invasive ductal carcinoma (up)	NUAK1 SPARC GEM SDC2 EFEMP2 LGALS1 COL15A1 COL8A2 PLN GREM1 ANGPTL2 DSE CALD1 RECK TGFB1I1 FAP WIPF1 KIAA1462 JAM3 MSN COL6A1 FGL2 CYR61 GIMAP4 RARRES2 SMAD7 MYL9 TMEFF1 SERPINF1 COL6A2 ECM2 CDH11 LHFP CD163 TRPC1 HMOX1 CTSK MAP1B COLEC12 WWTR1 MMP3 TGM2 AKT3 MXRA7 GFPT2 POSTN OLFML2B
M1	Cluster 11b urothelial carcinoma (up)	RUNX3 PDZRN3 TNC AP1S2 IL10RA MSN NUAK1 FHL1 MYL9 TNS1 DDR2 LEF1 SH2B3 COL6A2 SRPX CYR61 COLEC12 GEM TGFBR1 GSN CXCR4 SERPINF1 JAM3 CLIC4 CCL2 PCOLCE POSTN COL15A1 RARRES2 VCAM1 CXCL12 FGL2 LGALS1 GLIPR2 SPARC ITM2A PMP22 COL6A1 TPM2 ANGPTL2 ECM2
M1	Luminal vs mesenchymal breast cancer (down)	AKT3 DENND5A POPDC3 CD44 SACS CALD1 MXRA7 WIPF1 CDH11 MAP1B AP1S2 ATM FHL1 PMP22 HAS2 FLRT2 FOXC1 COL6A1 SPARC SRPX TGFB1I1 SH2B3 PRKCA COL6A2 LEPRE1 EFEMP2 SERPINE1 LHFP HMGA2 DSE GFPT2 WWTR1 MSN GLIPR2
M1	Luminal B breast cancer (down)	CLIC4 ENPP2 VCAM1 TSPAN8 CYR61 IGF1 MSN DPT CCL2 EDN1 FBLN5 WWTR1 GEM CXCL12 MAP1B TNC RUNX3 FHL1 GZMK CHRDL1 ELF5 MEOX2 ITM2A CALD1 SFRP4 PTGIS MMP3 TAGLN SRPX KIT FOXC1 HSPB2 MYCN MMP14 CRYAB
M1	Prostate cancer (down)	MUC4 DPT MYL9 KCNJ8 CHRDL1 FHL1 FGFR2 COL6A1 TNS1 CALD1 CHN1 MAP1B SRPX ANGPTL2 RARRES2 PMP22 TGFBR1 EFEMP2 SERPINF1 DDR2 CLIC4 TGFB1I1 KIT MXRA7 TPM2 CRYAB TUBA1A SPARC TAGLN LGALS1 DSE ITGB6 FBLN5
M1	Kras2La mouse lung cancer (down)	GUCY1B3 TCF3 KIT GSN CLIC4 POSTN RARRES2 FHL1 TMEFF1 FGL2 CDH11 CDK14 GIMAP4 PTGIS PMP22 CYR61 MYL9 ENPP2 DPT ANGPTL2 CXCL12 RECK TAGLN EDN1 CXCR4 MEOX2 WWTR1 COL6A2 SPARC TUBA1A
M1	Norma-like breast cancer (up)	CCL2 IGF1 CHRDL1 CXCL12 VCAM1 RUNX3 LEF1 FHL1 GEM F13A1 CXCL13 ENPP2 MEOX2 PLEKHO1 LHFP WIPF1 ITM2A CRYAB GIMAP4 SRPX GZMK DPT FBLN5 KIT SYNE1 FLRT2 HSPB2
M1	Papillary thyroid carcinoma (down)	ITM2A SYNE1 CHRDL1 FGL2 KIT DPT FLRT2 FHL1 PLN MYCN MEOX2 FBLN5 CXCL12 SDC2 CRYAB CYR61 BMP2 FGFR2 SRPX
M1	S1 hepatocellular carcinoma: WNT signalling	FGL2 CDH11 COL15A1 GEM DDR2 CXCR4 CHN1 TFF3 AKT3 POSTN MSN LGALS1 TPM2 COL6A2 TAGLN MAP1B COL6A1 CYR61 F13A1
M1	Invasive signature from microenvironemental interaction	GREM1 OLFML2B SFRP4 CDH11 COL6A2 CTSK SPARC LGALS1 POSTN NUAK1 PCOLCE FAP SERPINF1
M1	Advanced vs. early gastric cancer (up)	FAP SPARC NUAK1 GFPT2 DDR2 CDH11 CALD1 COL15A1 SFRP4 CD163 WWTR1 TRPC1 DSE CDK14 AKT3

M1	Ovarian tumor suboptimal debulk (up)	JAM3 COL15A1 RARRES2 CDK14 ITM2A STAT5B JAM2 ECM2 SIX1 TGFB1I1 SDC2 MYH14 PDZRN3 RECK TRPC1 ENPP2 SYNE1 KRT8 MEOX2
M1	Pancreatic ductal adenocarcinoma (up)	TFF3 MMP9 PMP22 SPARC MYL9 PLN CXCR4 GEM FAP CTSK POSTN SERPINE1 PCOLCE MSN TUBA1A CALD1
M1	Basal breast cancer (up)	TPM2 CXCL5 CLIC4 WWTR1 RUNX3 CRYAB MMP14 CCL2 SDC2 CD44 HSPB2 TMEFF1 FOXC1 MSN EDN1 CALD1 ELF5 KIT PRKCA
M1	Luminal vs basal breast cancer (down)	CLIC4 CD44 CALD1 DSE ITGB1 FHL1 AKT3 FAP CRYAB RUNX3 FGF2 WWTR1 SRPX MMP14 HMGA2 MSN
M1	Basal breast cancer (down)	PAX2 FAM174B ITM2A KRT8 CXCL12 TOX3 SFRP4 TFF3 MEOX2 IGF1 MLPH FBP1 F13A1 SIX1 ARHGAP32 CYB561 LEP TSPAN8 DPT
M1	Stromal co-expression in breast cancer	SERPINF1 CTSK FAP SPARC PCOLCE CDH11
M1	ADHC breast cancer (up)	MMP9 TNC CD163 CDH11 GREM1 FAP GAB2 SERPINE1 MMP3 VCAM1 CXCL13 GZMK CXCR4
M2	Invasive ductal carcinoma (up)	FYN DAB2 FBN1 LOX TCF4 BICC1 FN1 VCAN C1S RGS2 MAF DCN EMP3 ZEB1 MMP13 MMP2 SAMSN1 OLFML3 SPOCK1 C1R FSTL1 ZFPM2 COL5A2 SNAI2 HEG1 CTGF ZEB2
M2	Luminal vs mesenchymal breast cancer (down)	VIM BNC2 NR3C1 HEG1 DAB2 ENG RGS2 MCAM PTX3 FBN1 ZEB1 GJA1 SNAI2 EMP3 BICC1 SOAT1 FSTL1 PDE4A C1S CEP170 PDGFC PTGS2 MMP2
M2	Cluster 11b urothelial carcinoma (up)	MOXD1 C1S KLF4 MMP2 DAB2 C1R EMP3 MAF MCAM RGS2 FYN CTGF TCF4 COL5A2 SAMSN1
M2	Advanced vs. early gastric cancer (up)	VCAN FN1 DAB2 FSTL1 MOXD1 COL5A2 SPOCK1 PDGFC LOX FBN1 SNAI2 CEP170
M2	Luminal vs basal breast cancer (down)	C1S LOX STAT3 SOAT1 RGS2 BICC1 NR3C1 HIF1A PDGFC SNAI2 FSTL1 PTGS2 C1R EMP3 CDH13
M2	Invasive signature from microenvironemental interaction	VCAN COL5A2 SPOCK1 LOX MMP2 SNAI2 FN1 DCN FBN1
M2	Pancreatic ductal adenocarcinoma (up)	FBN1 PTGS2 ACVR1 KLF4 WNT5A EMP3 VCAN IGFBP5 FN1 COL5A2 FYN MMP2
M2	Mucinous ovarian carcinoma (down)	RUNX1T1 WNT5A TCF4 BNC2 DCN DAB2 ZFPM2 PDGFC C1S MAF
M2	Prostate cancer (down)	FYN C1R FBN1 PTGDS GJA1 SEMA3E HSPB1 MCAM C1S PDE4A SNAI2 PDGFC
M2	S1 hepatocellular carcinoma: WNT signaling	LOX DAB2 IGFBP5 TCF4 VCAN CTGF COL5A2 FBN1 HIF1A
M2	Luminal B breast cancer (down)	CDKN2A DCN FYN C1S CTGF SYNM RGS2 PTN PTGS2 VCAN PTX3
M2	Norma-like breast cancer (up)	TCF4 DCN FYN PTN C1S PTGDS SYNM C1R ZEB1 RUNX1T1
M2	Ovarian tumor suboptimal debulk (up)	CDH13 DCN SPOCK1 ZEB1 IGFBP5 RUNX1T1 PDE4A SNAI2 WNT5A GIPC2
M2	Serrated vs conventional colorectal cancer (up)	PDGFC C1S HIF1A TCF4 EMP3 CTGF
M2	Tumor methylation	CDKN2A CDH13 PTGS2 VCAN DAB2

M2	Kras2La mouse lung cancer (down)	KLF4 DCN LOX MPDZ TBX3 FYN ENG TCF4 RGS2
M3	Invasive ductal carcinoma (up)	SOBP GAS1 SERPING1 ASPN TMEM158 GLIPR1 FERMT2 GLRX TWIST1 MAFB SRGN S100A4 EDNRA ISLR BGN QKI AXL IGFBP3 EVI2A SPARCL1 PTRF PLXNC1
M3	Cluster 11b urothelial carcinoma (up)	TMEM158 SERPING1 EVI2A EDNRA AKAP12 PTRF EFEMP1 GLIPR1 GNG11 GPM6B SON MAPK7 MYLK SRGN ANPEP MAFB
M3	Advanced vs. early gastric cancer (up)	CYP1B1 TWIST1 IGFBP3 AXL EDNRA COL8A1 GAS1 EFEMP1 BGN MAFB QKI
M3	Luminal B breast cancer (down)	FBLN1 MYLK EFEMP1 GPM6B IGFBP3 FERMT2 WNT6 CYP1B1 MUC16 SRGN SERPING1 MMP7 MFAP4 GLIPR1 TMEM158
M3	Luminal vs mesenchymal breast cancer (down)	AKAP12 TWIST2 PTRF TGFB2 TMEM158 AXL GLIPR1 MYLK QKI SRGN ZCCHC24 TEAD1 TIMP1 GNG11
M3	Medullary vs ductal breast cancer (down)	PTRF ZCCHC24 EDNRA FERMT2 COL8A1 BGN PTPN14 MAPK7 MYLK
M3	Kras2La mouse lung cancer (down)	GPM6B SRGN MFAP4 QKI FERMT2 SERPING1 SPARCL1 GNG11 WISP2 AKAP12 PTRF
M3	Prostate cancer (down)	AKAP12 GPM6B TGFB2 PTRF MYLK FXYD6 EFEMP1 FGFR1 TGFB3 S100A4 FERMT2
M3	Basal breast cancer (down)	MFAP4 HNMT HGF ZCCHC24 FBLN1 BMP4 FGFR1 AR SPARCL1 WISP2 ASPN BMI1
M3	African-American vs. European-American (up)	GLRX TMEM158 EVI2A GNG11 CCL8 SRGN ANPEP EDNRA GLIPR1
M3	Norma-like breast cancer (up)	MYLK HGF SERPING1 GNG11 SPARCL1 EFEMP1 CYP1B1 MFAP4 GLIPR1 SRGN
M3	Mucinous ovarian carcinoma (down)	TGFB2 DDX5 FERMT2 EFEMP1 GAS1 COL8A1 EPB41L5
M3	Basal breast cancer (up)	PTPN14 GPM6B S100A4 WNT6 MMP7 CCL8 TMEM158 MYLK MUC16 QKI
M3	High grade prostatic intraepithelial neoplasia (up)	ASPN FBLN1 SPARCL1
M3	Circulating endothelial cells from cancer patients (up)	MAPK7 IGFBP3 MYLK CYP1B1 HNMT ANPEP
M3	Luminal A breast cancer (up)	MFAP4 FBLN1 ASPN PPARG WISP2
M3	CTNNB1 hepatocellular carcinoma (down)	TIMP1 BGN EVI2A EFEMP1 ASPN ISLR
M3	Prostate cancer vs. benign prostate tumor (down)	PTRF GAS1 MYLK AKAP12
M3	Papillary thyroid carcinoma (up)	CYP1B1 PLXND1 PLXNC1 COL8A1 S100A4 IGFBP3 TIMP1 MMP7
M3	Brain stem cell cancer signature	BGN FOXC2 S100A4 TEAD1
M3	Onocytic follicular vs mitochondrial rich papillary thyroid cancer (down)	RAF1 BGN TIMP1 COL8A1
M3	Invasive signature from microenvironemental interaction	ASPN BGN EDNRA TMEM158

Supplementary Table 8. List of primer sequences

Target gene	Forward primer	Reverse primer
CDH1	AAAGGCCCATTTCCTAAAAACCT	TGCGTTCTCTATCCAGAGGCT
SNAI1	TCGGAAGCCTAACTACAGCGA	AGATGAGCATTGGCAGCGAG
FN1	AGGAAGCCGAGGTTTTAACTG	AGGACGCTCATAAGTGTCACC
TWIST1	GTCCGCAGTCTTACGAGGAG	GCTTGAGGGTCTGAATCTTGCT
GRHL2	GGGGAAGAGCAACGAGTGG	GGGGAAGAGCAACGAGTGG
MMP3	CTGGACTCCGACACTCTGGA	CAGGAAAGGTTCTGAAGTGACC
VIM	GACGCCATCAACACCGAGTT	CTTTGTCGTTGGTTAGCTGGT
GAPDH	GAAGGTGAAGGTCGGAGT	GAAGATGGTGATGGGATTTC
ACTB	CTTCTACAATGAGCTGCGTG	GGGTGTTGAAGGTCTCAAAC

Supplementary Table 9. Parameter values for mathematical models

Parameter	Description	Value
$\gamma_{\mathrm{TGF\beta}}$	Timescale of TGF-β	1
γ_{ZEB1}	Timescale of ZEB1	1
γ_{X}	Timescale of factor X (e.g. OVOL2, miR200)	1
γ_{M1}	Timescale of M1 genes	1
γ_{M2}	Timescale of M2 genes	1
γ_{M3}	Timescale of M3 genes	1
γ_{E}	Timescale of E genes	1
$\sigma_{\mathrm{TGF\beta}}$	Steepness of sigmoidal function of TGF-β	10
σ_{ZEB1}	Steepness of sigmoidal function of ZEB1	10
σ_{X}	Steepness of sigmoidal function of factor X	10
σ_{M1}	Steepness of sigmoidal function of M1 genes	10
σ_{M2}	Steepness of sigmoidal function of M2 genes	10
σ_{M3}	Steepness of sigmoidal function of M3 genes	10
σ_{E}	Steepness of sigmoidal function of E genes	10
$w^0_{\mathrm{TGF\beta}}$	Basal production rate of TGF-β	-1
w^0_{ZEB1}	Basal production rate of ZEB1	0.2
w^0_{X}	Basal production rate of factor X	0.5
w^0_{M1}	Basal production rate of M1 genes	-0.5
w^0_{M2}	Basal production rate of M2 genes	-0.5
w^0_{M3}	Basal production rate of M3 genes	-0.5
w^0_{E}	Basal production rate of E genes	0.5
$\omega_{\mathrm{TGF\beta \to ZEB1}}$	Activation strength of TGF-β on ZEB1	1
$\omega_{\mathrm{ZEB1 \to TGF\beta}}$	Activation strength of ZEB1 on TGF-β	1
$\omega_{\mathrm{ZEB1 \to X}}$	Inhibition strength of ZEB1 on factor X	-0.6
$\omega_{\mathrm{X \to ZEB1}}$	Inhibition strength of X on factor ZEB1	-0.6
$\omega_{\mathrm{ZEB1 \to E}}$	Inhibition strength of ZEB1 on E genes	-1
$\omega_{\mathrm{TGF\beta \to M1}}$	Activation strength of TGF-β on M1 genes	0.5*
$\omega_{\mathrm{ZEB1 \to M1}}$	Activation strength of ZEB1 on M1 genes	0.1**
$\omega_{\mathrm{TGF\beta \to M2}}$	Activation strength of TGF-β on M2 genes	1
$\omega_{\mathrm{ZEB1 \to M2}}$	Activation strength of ZEB1 on M2 genes	1
$\omega_{\mathrm{ZEB1 \to M3}}$	Activation strength of ZEB1 on M3 genes	0.7

* Total activation is saturated at 0.4   ** Total activation is saturated at 0.1

SUPPLEMENTARY REFERENCES

1 Taube, J. H. *et al.* Core epithelial-to-mesenchymal transition interactome gene-expression signature is associated with claudin-low and metaplastic breast cancer subtypes. *Proc. Natl. Acad. Sci. U. S. A.* **107**, 15449-15454, doi:10.1073/pnas.1004900107 (2010).

2 Huber, W. *et al.* Orchestrating high-throughput genomic analysis with Bioconductor. *Nat. Methods* **12**, 115 (2015).

3 Jolly, M. K., Ware, K. E., Gilja, S., Somarelli, J. A. & Levine, H. EMT and MET: necessary or permissive for metastasis? *Mol. Oncol.* **11**, 755-769 (2017).

4 Subramanian, A. *et al.* Gene set enrichment analysis: a knowledge-based approach for interpreting genome-wide expression profiles. *Proc. Natl. Acad. Sci. USA* **102**, 15545-15550 (2005).

5 Barretina, J. *et al.* The Cancer Cell Line Encyclopedia enables predictive modelling of anticancer drug sensitivity. *Nature* **483**, 603 (2012).
